# Inhalation of printer-emitted particles impairs cardiac conduction, hemodynamics, and autonomic regulation and induces arrhythmia and electrical remodeling in rats

**DOI:** 10.1186/s12989-019-0335-z

**Published:** 2020-01-29

**Authors:** Alex P. Carll, Renata Salatini, Sandra V. Pirela, Yun Wang, Zhengzhi Xie, Pawel Lorkiewicz, Nazratan Naeem, Yong Qian, Vincent Castranova, John J. Godleski, Philip Demokritou

**Affiliations:** 1 Department of Physiology, School of Medicine, University of Louisville, Louisville, KY USA; 2 Christina Lee Brown Envirome Institute, University of Louisville, Louisville, KY USA; 3 Center for Nanotechnology and Nanotoxicology. Department of Environmental Health, T.H. Chan School of Public Health, Harvard University, 665 Huntington Avenue, Room 1310, Boston, MA 02115 USA; 4 Department of Surgery, University of Sao Paulo Medical School, Sao Paulo, Brazil; 5 Department of Occupational and Environmental Health Sciences,School of Public Health, Peking University, Beijing, People’s Republic of China; 6 Pathology and Physiology Research Branch, Health Effects Laboratory Division, National Institute for Occupational Safety and Health, Morgantown, WV USA; 7 Department of Pharmaceutical Sciences/Mary Babb Randolph Cancer Center, West Virginia University, Morgantown, WV USA

**Keywords:** Arrhythmia, Particulate matter, Autonomic, Electrocardiogram, Cardiac, Nanoparticles, Printers, Telemetry, Left ventricular pressure, Contractility, Repolarization, Electrical remodeling

## Abstract

**Background:**

Using engineered nanomaterial-based toners, laser printers generate aerosols with alarming levels of nanoparticles that bear high bioactivity and potential health risks. Yet, the cardiac impacts of printer-emitted particles (PEPs) are unknown. Inhalation of particulate matter (PM) promotes cardiovascular morbidity and mortality, and ultra-fine particulates (< 0.1 μm aerodynamic diameter) may bear toxicity unique from larger particles. Toxicological studies suggest that PM impairs left ventricular (LV) performance; however, such investigations have heretofore required animal restraint, anesthesia, or ex vivo preparations that can confound physiologic endpoints and/or prohibit LV mechanical assessments during exposure. To assess the acute and chronic effects of PEPs on cardiac physiology, male Sprague Dawley rats were exposed to PEPs (21 days, 5 h/day) while monitoring LV pressure (LVP) and electrocardiogram (ECG) via conscious telemetry, analyzing LVP and heart rate variability (HRV) in four-day increments from exposure days 1 to 21, as well as ECG and baroreflex sensitivity. At 2, 35, and 70 days after PEPs exposure ceased, rats received stress tests.

**Results:**

On day 21 of exposure, PEPs significantly (*P* < 0.05 vs. Air) increased LV end systolic pressure (LVESP, + 18 mmHg) and rate-pressure-product (+ 19%), and decreased HRV indicating sympathetic dominance (root means squared of successive differences [RMSSD], − 21%). Overall, PEPs decreased LV ejection time (− 9%), relaxation time (− 3%), *tau* (− 5%), RMSSD (− 21%), and P-wave duration (− 9%). PEPs increased QTc interval (+ 5%) and low:high frequency HRV (+ 24%; all *P* < 0.05 vs. Air), while tending to decrease baroreflex sensitivity and contractility index (− 15% and − 3%, *P* < 0.10 vs. Air). Relative to Air, at both 2 and 35 days after PEPs, ventricular arrhythmias increased, and at 70 days post-exposure LVESP increased. PEPs impaired ventricular repolarization at 2 and 35 days post-exposure, but only during stress tests. At 72 days post-exposure, PEPs increased urinary dopamine 5-fold and protein expression of ventricular repolarizing channels, K_v_1.5, K_v_4.2, and K_v_7.1, by 50%. Conclusions: Our findings suggest exposure to PEPs increases cardiovascular risk by augmenting sympathetic influence, impairing ventricular performance and repolarization, and inducing hypertension and arrhythmia. PEPs may present significant health risks through adverse cardiovascular effects, especially in occupational settings, among susceptible individuals, and with long-term exposure.

## Background

Cardiovascular disease (CVD) is the leading global cause of mortality, and among its primary risk factors are high blood pressure and exposure to air pollution [1]. Among air pollutants, particulate matter (PM) is most consistently tied to increased cardiovascular morbidity and mortality. Globally, household air pollution causes an estimated 2.8 million deaths, and exposures to ambient PM account for 4.2 million deaths per year, 57% of which are cardiovascular in origin [2]. Multiple, often interacting, modes of action underlie the cardiovascular toxicity of PM, including enhanced sympathetic regulation, arrhythmia, oxidative stress, inflammation, vascular dysfunction, and exacerbation of both atherosclerosis and heart failure [3]. Modern sources of indoor air pollution may pose important health risks, especially in industrialized countries, where adults now spend ≈90% of their time indoors [4].

Engineered nanomaterials (ENMs), which have at least one dimension in the nanoscale (1–100 nm), are synthesized and used across several scientific fields and in various cosmetics, food, building materials, and medicines. Exposures to ENMs released across the lifecycle of nano-enabled products have become inevitable. Due to their size, ENMs can bypass biological barriers, become systemic, interfere with cellular processes, and induce adverse health effects [5–15]. Despite that numerous studies have linked exposure to ambient ultrafine particles to cardiovascular dysfunction, autonomic dysregulation, and heart disease [16–19], there are only limited investigations into the cardiovascular effects of ENMs [20].

Laser printer toners are nano-enabled products widely used in office and household microenvironments [21, 22]. The authors and others have performed thorough physico-chemical and toxicological characterizations of laser printer and photocopier-emitted PM [8, 21–30], whose complex chemical makeup includes toxic constituents such as transition metals (e.g., zinc, chromium, nickel, iron, titanium, and aluminum), volatile organic chemicals (VOCs), and polycyclic aromatic hydrocarbons (PAHs). Exposures to this class of PM may lead to adverse health outcomes, as worksites with high print volumes often have indoor PM concentrations far exceeding the advised limits for ambient PM_2.5_ [21]. Our recent work indicates that exposure to PEPs promotes airway inflammation and microvascular remodeling [21, 28]. However, the cardiovascular effects of PEPs remain unexplored.

Exposures to PM aerosols can impair LV systolic performance, indicated by decreases in ejection fraction, fractional shortening, and—assuming unaltered systolic and diastolic pressures—maximum LV pressure slope (*dP/dt*_max_) [31–35]. Declines in these markers, along with LV ejection time [36, 37] and contractility index (pressure-normalized *dP/dt*_max_) [35], reflect diminished LV contractility and can denote heart failure: an inability of the LV to perfuse vital tissues. PM exposure can also impede ventricular repolarization, seen on the ECG as prolonged QT and T_peak_-T_end_ (TpTe) [38–45], a phenotype so predictive of arrhythmia and sudden cardiac death that its appearance has banished countless pharmaceuticals from the market [46–49]. As PM and other PEPs constituents (e.g., Ni, Fe, VOCs, and PAHs) are associated with impaired ventricular contractility, heart failure, electrophysiologic defects, and arrhythmia [3, 33, 50, 51], we sought to determine the impacts of PEPs exposures on cardiac function. Real-time LVP and ECG were continuously monitored in conscious unrestrained rats during and after whole body inhalation exposure to PEPs. We hypothesized that a 21-day (5 h per day) exposure to PEPs would impair LV performance, induce autonomic imbalance, and impede cardiac conduction.

To the best of our knowledge, no study has yet examined cardiac mechanical function in conscious animals *during* inhalation exposure to an air pollutant, much less ENMs like PEPs that are released across the lifecycle of nano-enabled products. Indeed, effects of air pollutants on cardiac mechanical function have been examined *after* exposures upon the restoration of clean air, but post-exposure assessments may allow effects to subside with compensatory responses and/or dissipation of irritant reflexes, especially with exposures more representative of environmental concentrations. Thus, to provide the first ever assessment of conscious LV performance *during* a pollutant aerosol exposure, we analyzed LV systolic and diastolic function both during and after PEPs exposure in conscious un-restrained rats. Further, to unmask latent and persistent cardiac effects, at 2, 35, and 70 days following cessation of inhalation exposures to PEPs, we incorporated an acute stress-test known to markedly increase blood pressure, heart rate, and catecholamines in rats [52].

## Results

### Characterization of PEPs exposure

Rats were exposed to PEPs in whole-body inhalation exposure chambers as described in detail by the authors in previous publications and summarized in the Methods section below [27, 53]. An empty exposure chamber was sampled continuously throughout the study for aerosol characterization. The mean concentration of PEPS across the 21-day exposure was 0.498 million particles/cm^3^ by count and 71.5 μg/m^3^ by mass (Table 1). These concentrations are within the range found in exposure assessments in printing equipment facilities around the world. For instance, our previous investigation of 8 copier centers in the greater Boston area (USA) found weekly mean nanoparticle number concentrations reaching approximately 12 times higher than background levels (before start of printing activity), with maximum temporal emission recorded at 700-fold higher than the background average and at levels exceeding 1,000,000 particles/cm^3^ [30]. In the current study, PEPs size distributions were relatively constant across the 21-day exposure period, with daily count median diameters ranging from 39.2 to 48.9 nm. The identified particle size distribution of PEPs was consistent throughout the exposure, with geometric standard deviation (GSD) values almost unchanged ranging from 1.65 to 1.86, with a mean of 1.71. Additional file 1: Figure S1 summarizes the particle number concentration as a function of size. The complex chemical composition of PEPs has been characterized in great detail in prior publications. Previous studies by our group have shown that printer B1 emits up to 1.3 million particles/cm^3^ of varying mobility diameters ranging from 33 to 43 nm using the same printing protocol as in this study [27]. Further, the PEPs emitted by printer B1 under the same protocol are composed of a complex mixture of 97% organic carbon, 0.5% elemental carbon, and 2.5% metals (Al, Fe, Cu, and Si) [22]. Further, organic compounds on PEPs included both low and high molecular weight carcinogenic PAHs which are the result of interactions of catalytic metal and metal oxide nanoparticles with emitted semi-volatile organic compounds [22, 23]. The total volatile organic gaseous compounds (tVOCs) were also measured and found at low concentrations, with daily averages between 245 ± 164 parts per billion (ppb) and 363 ± 162 ppb [22, 23].

**Table 1:** PEPs aerosol concentrations

	Mean	SD	Min	Max
Number concentration (#/cm^3^)	497,569	96,499	338,818	684,640
Mass concentration (μg/m^3^)	71.5	15.6	43.3	108.4
Mean mobility diameter (nm)	49.5	2.5	44.5	53.2
Count median diameter (nm)	44.6	2.6	39.2	48.9
Geom. Standard deviation (nm)	1.71	0.04	1.65	1.86

Mean, standard deviation (SD), minimum (min), and maximum (max) daily values over the 21-day exposure

#### Physiology before exposure

**Fig. 1:**
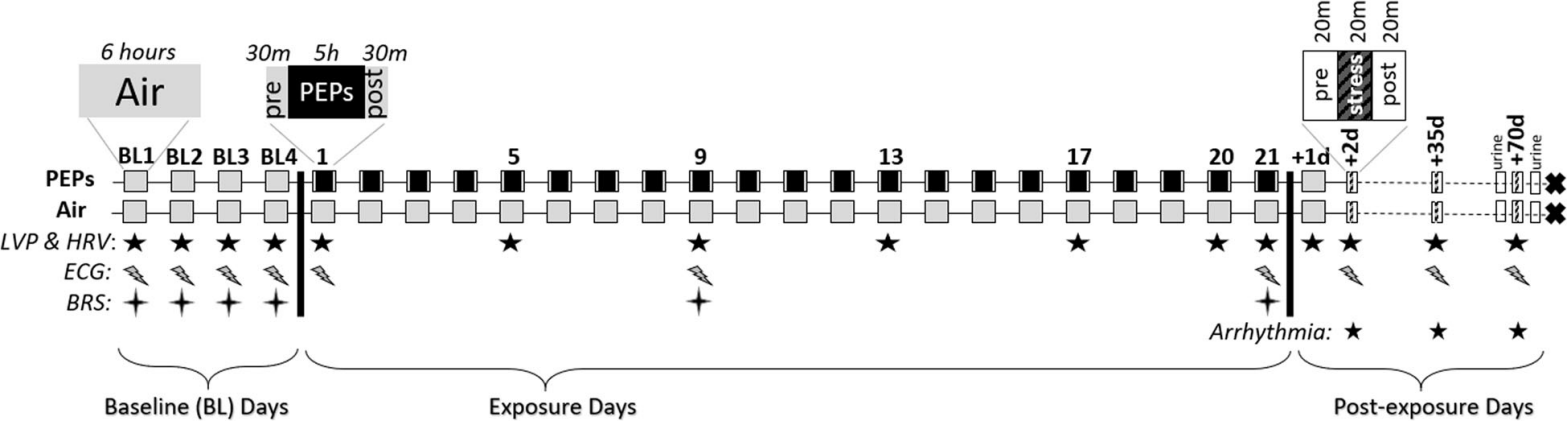
Exposure and analysis timeline. Gray boxes mark control exposures to HEPA-filtered air for six hours per day, including four successive BL (BL) days. Black boxes with gray borders indicate PEPs exposures preceded and followed by 30-min clean air exposures. White boxes indicate post-exposure sampling periods, with striped boxes marking 20-min stress tests at 2 days, 5 weeks, and 10 weeks after cessation of inhalation exposures. Empty boxes mark urine collection periods before and after stress test at 10 weeks post-PEPs. Stars indicate period during which physiologic endpoints were analyzed, including left ventricular pressure (LVP), heart rate variability (HRV), electrocardiogram morphology (ECG), baroreflex slope (BRS), and ventricular arrhythmia. For more details, see *Methods*

All rats received clean filtered air in exposure chambers for 6 h each day over four successive baseline (BL) days preceding PEPs aerosol generation (Fig. 1). During BL, LVP indices and ECG morphology did not differ between the groups designated for subsequent air or PEPs exposures (Additional file 1: Table S1). The standard deviation of normal RR intervals (SDNN) was 45% higher in the PEPs group, indicating higher HRV in this group. Measures of ventricular repolarization, including uncorrected QT (measured to T_end_) and TpTe, were comparable to historic values from male Sprague Dawley rats of the same age and sampling conditions but lacking LV catheterization (LV catheterized vs. non-catheterized rats with ECG telemetry, mean ± SEM QT = 55.9 ± 2.1 ms vs. 56.5 ± 0.8 ms; TpTe = 25.5 ± 1.8 ms vs. 28.1 ± 1.6 ms).

#### Autonomic and cardiac effects during exposure

**Fig. 2:**
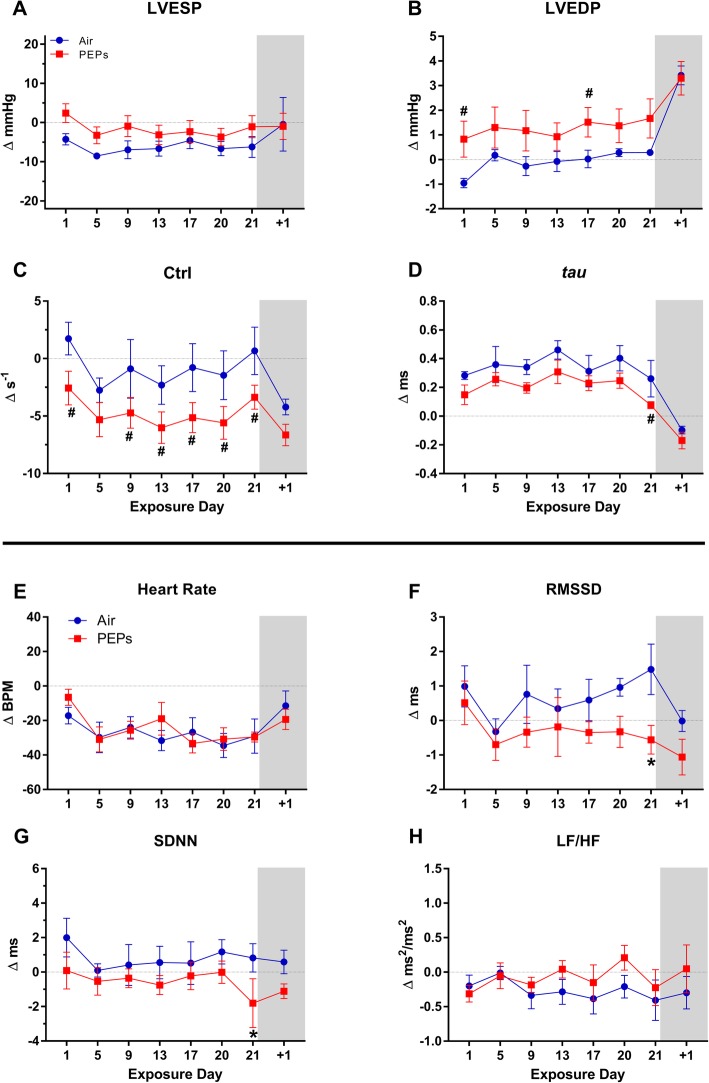
Change from BL in LVP and HRV during exposure. Values calculated as mean (± standard error) of each animal’s change from its 4-day BL (5 h/day). For LVP (**a-d**), Air *n* = 3 and PEPs *n* = 4. For HRV (**e-h**), *n* = 4/group. Day + 1 marks post-exposure day in ambulatory monitoring cages. ^#^*P* < 0.10 and *P < 0.05 vs. Air. BL means ± SEM for Air and PEPs groups, respectively: LVESP = 121.7 ± 1.4 and 115.7 ± 1.9 mmHg; LVEDP = 3.6 ± 0.5 and 3.2 ± 0.9 mmHg; CtrI = 114.6 ± 0.8 and 114.6 ± 2.0 s^− 1^; *tau* = 6.7 ± 0.1 and 6.8 ± 0.1 ms; heart rate = 333 ± 4 and 332 ± 6 BPM; RMSSD = 3.00 ± 0.34 and 4.68 ± 0.46 ms; SDNN = 8.10 ± 0.40 and 11.74 ± 0.71 ms; and LF/HF = 1.06 ± 0.14 and 1.28 ± 0.15

**Fig. 3:**
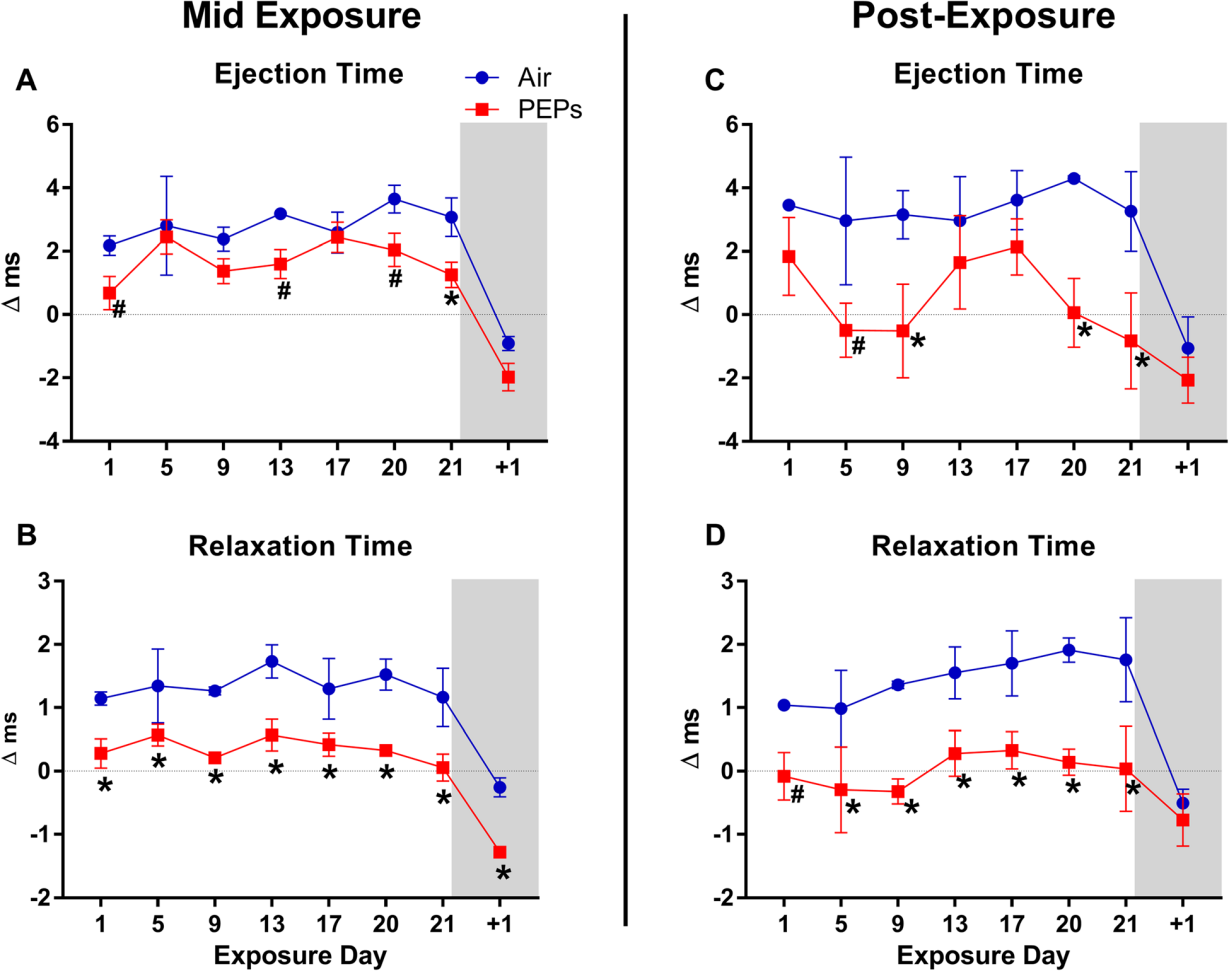
Change in LV relaxation and ejection times during (**a**-**b**) and immediately after (**c**-**d**) aerosol exposure. Day + 1 marks post-exposure day in ambulatory monitoring cages instead of exposure chambers. Air *n* = 3 and PEPs *n* = 4. Values calculated as mean (± standard error) of each animal’s change from its 4-day BL (mid-expo: 5 h/day, post-expo: 30 min/day). ^#^*P* < 0.10 and *P < 0.05 vs. Air. BL means ± SEM for Air and PEPs groups, respectively: Ejection Time = 30.9 ± 0.5 and 31.3 ± 0.6 ms; and Relaxation Time = 47.4 ± 0.3 and 48.3 ± 0.3 ms

LVP and HRV were analyzed on all BL days and one third of exposure days (7 of 21 days, Fig. 1). During exposure overall, PEPs decreased the root means squared of successive RR interval differences (RMSSD), a time-domain HRV parameter that denotes relative parasympathetic influence over the heart, compared to the Air group (Table 2, *P* < 0.05). Overall, PEPs decreased ejection time (EjeT, Table 2; *P* < 0.05 vs. Air), an index of contractility that is load independent and especially sensitive to cardiac myosin activation [37], and was associated with an overall trend of decreased contractility index (CtrI, Table 2; *P* < 0.10 vs. Air). Collectively, these effects suggest impairments in LV contractility during exposure to PEPs. Conversely, PEPs decreased relaxation time (RT), an inverse index of diastolic performance [35], suggesting augmented diastolic function. Across the three exposure days analyzed for ECG morphology (Fig. 1), PEPs significantly decreased S amplitude (S_amp_) and P-duration (P_dur_) overall (Table 1), suggesting accelerated atrial depolarization relative to Air (*P* < 0.05). On individual days of exposure, effects on LV performance and autonomic balance were complementary to the aforementioned effects overall (Figs. 2a-h and 3a-b). Most notably, on the final exposure day, PEPS significantly decreased HRV (SDNN and RMSSD (Fig. 2f-g), HF [Additional file 1: Figure S2], and additional variables [Additional file 1: Table S2]), and decreased EjeT and RelT (Fig. 3a-b), suggesting diminished contractility despite increased sympathetic regulation and diastolic function. Concurrently, PEPs exposure corresponded with a trend of decreased CtrI, a load -independent marker of contractility (Fig. 2c and Additional file 1: Table S2; *P* < 0.10 vs. Air). Notably, trends of decreased CtrI occurred during six of the seven analyzed PEPs exposures (Fig. 2c). RT was decreased on each PEPs day compared to Air (Fig. 3b, *P* < 0.05), and positively correlated across both exposure groups with changes in HRV, including RMSSD (Pearson’s *r* = 0.55), SDNN (*r* = 0.47), and high frequency (HF, *r* = 0.39, all *P* < 0.05), indicating the PEPs augmented diastolic function in concert with sympathetic influence. Interestingly, daily CtrI values also positively correlated with HRV, but only among rats in the Air group (RMSSD, Pearson’s *r* = 0.51; SDNN *r* = 0.41; HF *r* = 0.47; LF/HF *r* = − 0.63; all *P* < 0.05), whereas PEPs abolished this relationship (all *P* > 0.05), indicating PEPs disrupted the link between basal autonomic regulation and inotropy. Except for a depression in RT, PEPs-induced effects vanished 1 day after the 21-day exposure (day + 1), during ambulatory monitoring (in cages with bedding, room for movement, and food), when HR and EDP similarly increased in both groups.

**Table 2:** Overall effects of PEPs on LVP, HRV, and ECG morphology before, during, and immediately after inhalation exposures and stress tests

			Inhalation Exposure		Stress Test		
			Mid-Expo	Post-Expo	Pre-Stress	Mid-Stress	Post-Stress
LV P	Δ from BL	ESP (mmHg)^a^	–	9.8 ± 5.1	6.5 ± 2.5*	–	–
		CtrI (s^−1^)^a^	−3.9 ± 2.2	–	–	–	–
		Ejection Time (ms)^b^	−1.1 ± 0.5*	−2.8 ± 1.1*	−3.3 ± 1.7	−5.4 ± 2.1*	−4.6 ± 2.4
		*tau* (ms)^a^	–	−0.31 ± 0.13*	−0.34 ± 0.17	–	–
		Relaxation Time (ms)^b^	−1.0 ± 0.2*	−1.5 ± 0.4*	–	–	−2.2 ± 0.7*
		*dP/dt*_max_ (mmHg/s)^a^	–	–	692 ± 260*	–	609 ± 267*
		*dP/dt*_min_ (mmHg/s)^a^	–	− 628 ± 343	− 613 ± 263*	–	− 666 ± 224*
		devP (mmHg)^a^	–	–	–	–	8.7 ± 3.6*
		No overall differences in EDP					
HRV & BRS	Δ from BL	HR (beats/min)	–	–	21 ± 10	–	–
		BRS (ms/mmHg)	−0.32 ± 0.19	–			
		RMSSD (ms)	−1.0 ± 0.5*	−1.2 ± 0.6	–	–	–
		LF/HF	–	0.31 ± 0.15*	–	–	–
		No overall differences in SDNN, LF, and HF					
ECG Morphology	Δ from BL	Pdur (ms)	−1.7 ± 0.6*	−1.5 ± 0.5*	–	–	–
		QT (ms)^c^	–	1.4 ± 0.7	–	–	–
		QTc (ms)^c^	–	3.0 ± 1.2*	–	5.4 ± 2.6	–
		S amplitude (mV)	−0.031 ± 0.014*	–	–	–	–
		No overall differences in PR, QRS, STneg area, and T amplitude					
	Δ from Pre-stress	QT (ms)				8.1 ± 2.9*	–
		QTc (ms)				9.8 ± 2.5*	4.5 ± 1.9*
		ST neg area (ms*mv)				–	0.18 ± 0.8*
		T amplitude (mV)				0.052 ± 0.017*	0.037 ± 0.013*
		TpTe (ms)				9.1 ± 4.2*	–
		TpTe/QT (%)				8.5 ± 2.2*	–

Parameters were assessed as animal-matched change from the average of a 4-day BL exposure to clean air, or as animal-matched change from 20-min pre-stress period immediately before each of three stress tests. *n* = 4/group except ^a^*n* = 3 for Air group, and ^b^*n* = 3 for Air group during inhalation exposures only

^c^Interval terminus measured as T-peak

Effect estimates are presented as difference from time-matched Air control where *P* < 0.10, with * denoting significant difference from Air group (*P* < 0.05). “- “denotes *P* > 0.10

#### Autonomic and cardiac effects early after exposure

**Fig. 4:**
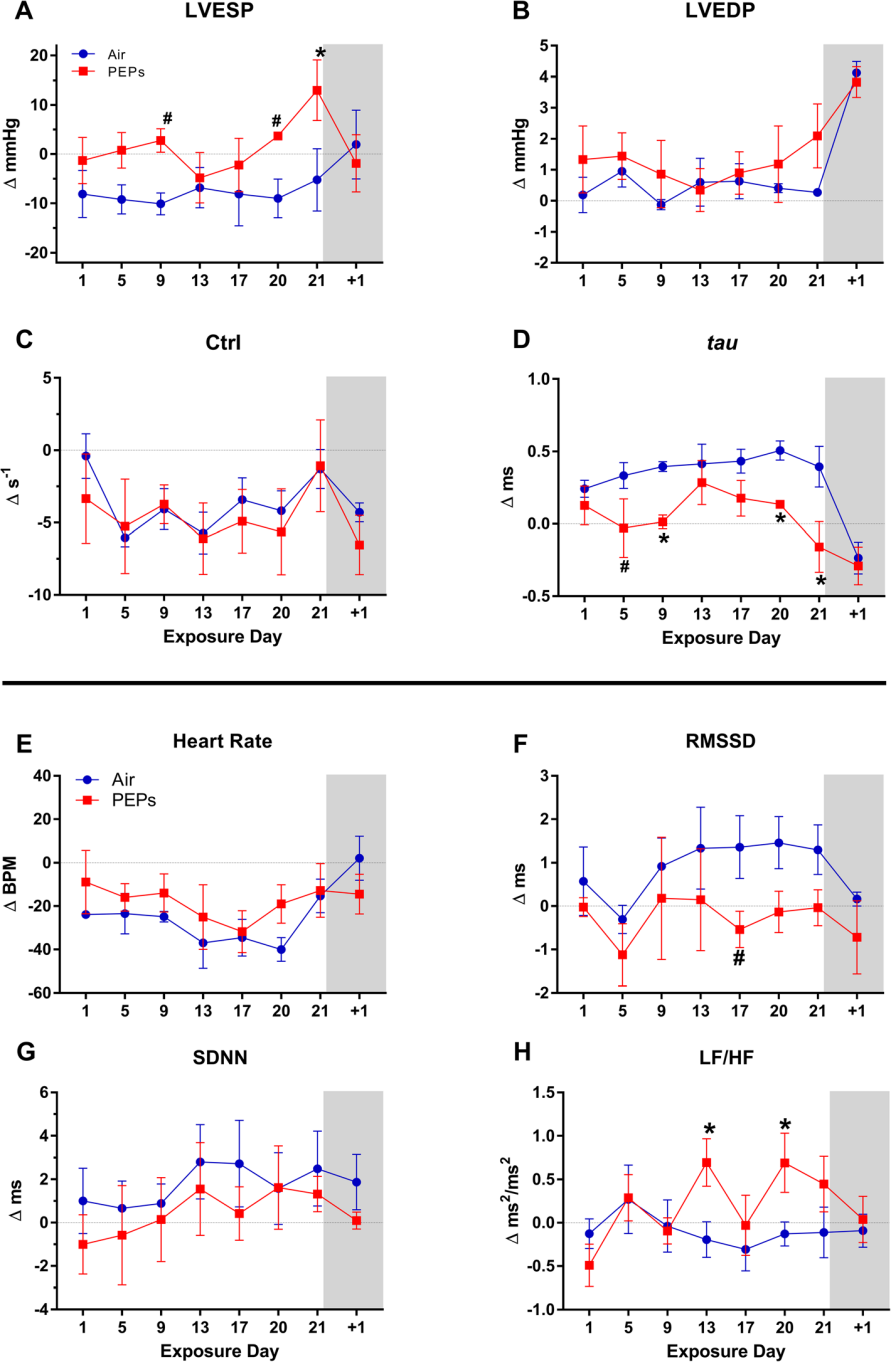
Change from BL in LVP and HRV immediately after exposure. Values calculated as mean (± standard error) of each animal’s change from its 4-day BL (30 min/day). For LVP (**a-d**), Air *n* = 3 and PEPs *n* = 4. For HRV (**e-h**), *n* = 4 / group. Day + 1 denotes post-exposure day in ambulatory monitoring cages. ^#^*P* < 0.10 and *P < 0.05 vs. Air. See Fig. 2 caption or Table S1 for BL means

During the 30-min post-exposure phase of each analysis day (Fig. 1), PEPs increased low to high frequency ratio (LF/HF) overall, suggesting sympathetic dominance. Concurrently, PEPs decreased EjeT and *tau* overall (Table 2), suggesting diminished contractility but augmented lusitropy. PEPs exposure was also associated with a significant prolongation of QTc (*P* < 0.05 vs. Air, Table 2), suggesting impaired ventricular repolarization. When analyzed for day-specific effects at the post-exposure phase, exposure day 21 had the most effects on LVP, including marked increases in LVESP, RPP, and *dP/dt*_max_, consistent with hypertension, and decreases in *dP/dt*_min_, *tau*, EjeT, and electro-mechanical coupling (EMC) that suggested diminished contractility despite enhanced lusitropy and excitation-contraction coupling (Figs. 2 and 3, Additional file 1: Figure S3 and Table S2, all *P* < 0.05 vs. Air). On day 9 both *tau* and EjeT were significantly decreased at post-exposure (Figs. 3c and 4d; *P* < 0.05 vs. Air), concomitant with a trend of increased LVESP (Fig. 4a, + 12.9 mmHg, *P* < 0.10 vs. Air). On day 20, EjeT was also significantly decreased after PEPs exposure (Fig. 3c). Notably, *tau* at post-treatment significantly correlated with concurrent RMSSD (Pearson’s *r* = 0.54, *P* < 0.0001) and HF (*r* = 0.41, *P* = 0.002), consistent with sympatho-excitation enhancing diastolic function. In addition to day 21, PEPs significantly increased RPP on day 20 (Additional file 1: Figure S3), which was further consistent with sympathetic dominance.

#### Effects on BRS

Because PEPs increased LVESP on exposure day 21 and was associated with a similar trend on day 9, spontaneous baroreflex sensitivity (BRS) was assessed on these days as well as BL days (Fig. 1). In contrast with BRS slope in the control group, which was strikingly consistent with BL, PEPs tended to decrease BRS slope during exposure overall (*P* < 0.10, Table 2) and diminished BRS on each individual day despite not reaching statistical significance (Additional file 1: Figure S4). Notably, daily BRS across both groups correlated strongly with HRV during the exposure phase (RMSSD *r* = 0.64; SDNN *r* = 0.56; HF *r* = 0.68; LF *r* = 0.71; all *P* < 0.05) and post-exposure phase (RMSSD *r* = 0.69; SDNN *r* = 0.73; HF *r* = 0.71; LF *r* = 0.71; all *P* < 0.05), indicating BRS positively correlated with parasympathetic modulation of the heart.

#### Long-term effects on resting cardiovascular physiology

**Fig. 5:**
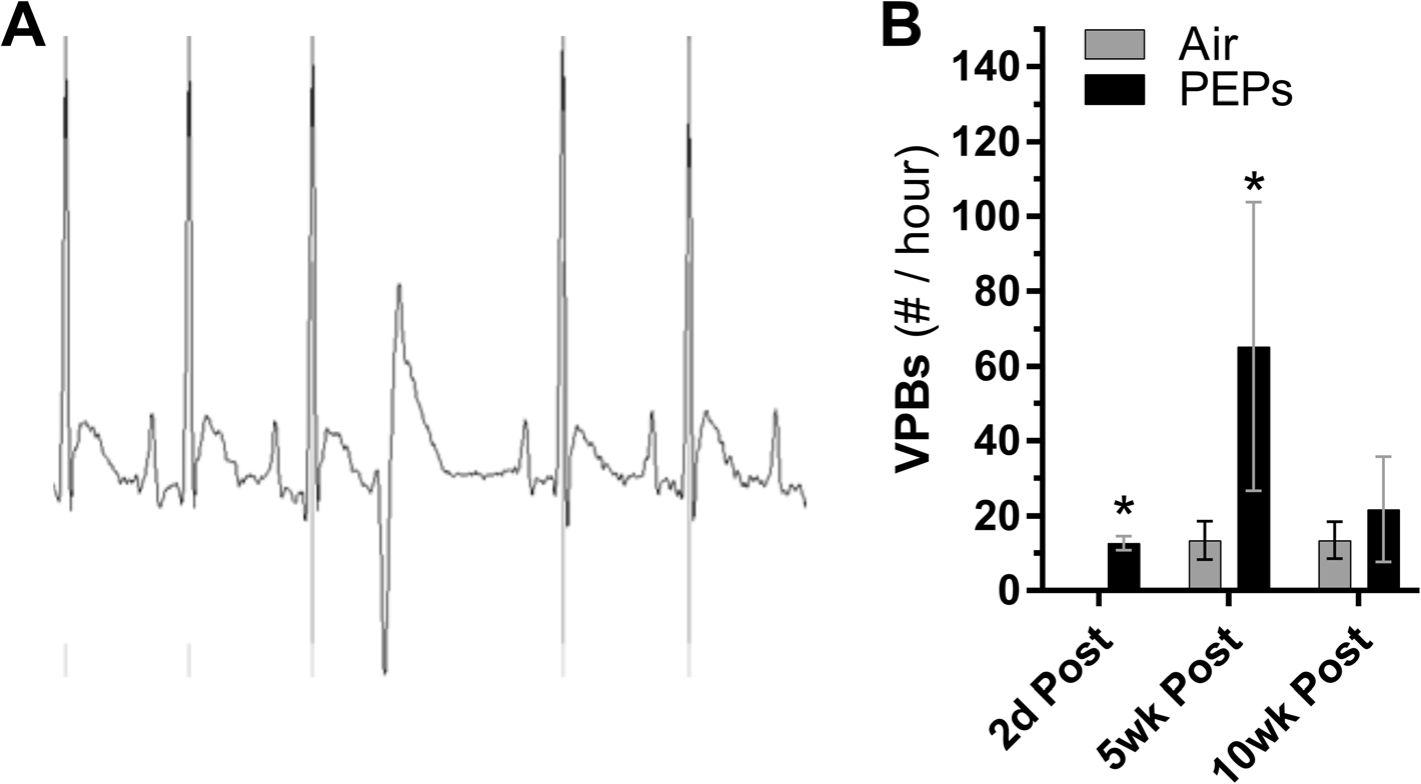
Spontaneous ventricular premature beats (VBPs) after a 21-day inhalation exposure to PEPs. **a**, representative VPB in a PEPs- exposed rat. **b**, frequency of VPBs among rats exposed to either filtered air or PEPs for 21 days. Values expressed as mean (± SEM) count of VPBs per hour during 20-min ambulatory observation period. *N* = 4/group. ^#^*P* < 0.10 and *P < 0.05 vs. Air

Rats were sampled during 20-min resting periods before stress tests at 2 days, 5 weeks, and 10 weeks post-exposure (Fig. 1). Overall, the PEPs group had significantly increased LVESP and *dP/dt*_max_ and decreased *dP/dt*_min_ relative to Air (Table 2). At 2 days post-exposure, PEPs significantly increased spontaneous ventricular tachyarrhythmias (Fig. 5b) and prolonged basal LV Filling Time (Additional file 1: Table S3). PEPs continued to increase resting VPBs at 5 weeks post-exposure, when it also increased basal *dP/dt*_max_ and decreased basal *dP/dt*_min_. At 10 weeks post-exposure, PEPs significantly increased LVESP and *dP/dt*_max_ and decreased RT, *dP/dt*_min_, and *tau* (Additional file 1: Table S3; *P* < 0.05 vs. Air). Mean basal HR (±SE) was 318 ± 7, 309 ± 4, and 300 ± 4 BPM for the Air group, respectively, at day 2, week 5, and week 10 of post-exposure, with no differences from the PEPs group (Additional file 1: Figure S5).

#### Long-term effects on cardiovascular responses to stress

**Fig. 6:**
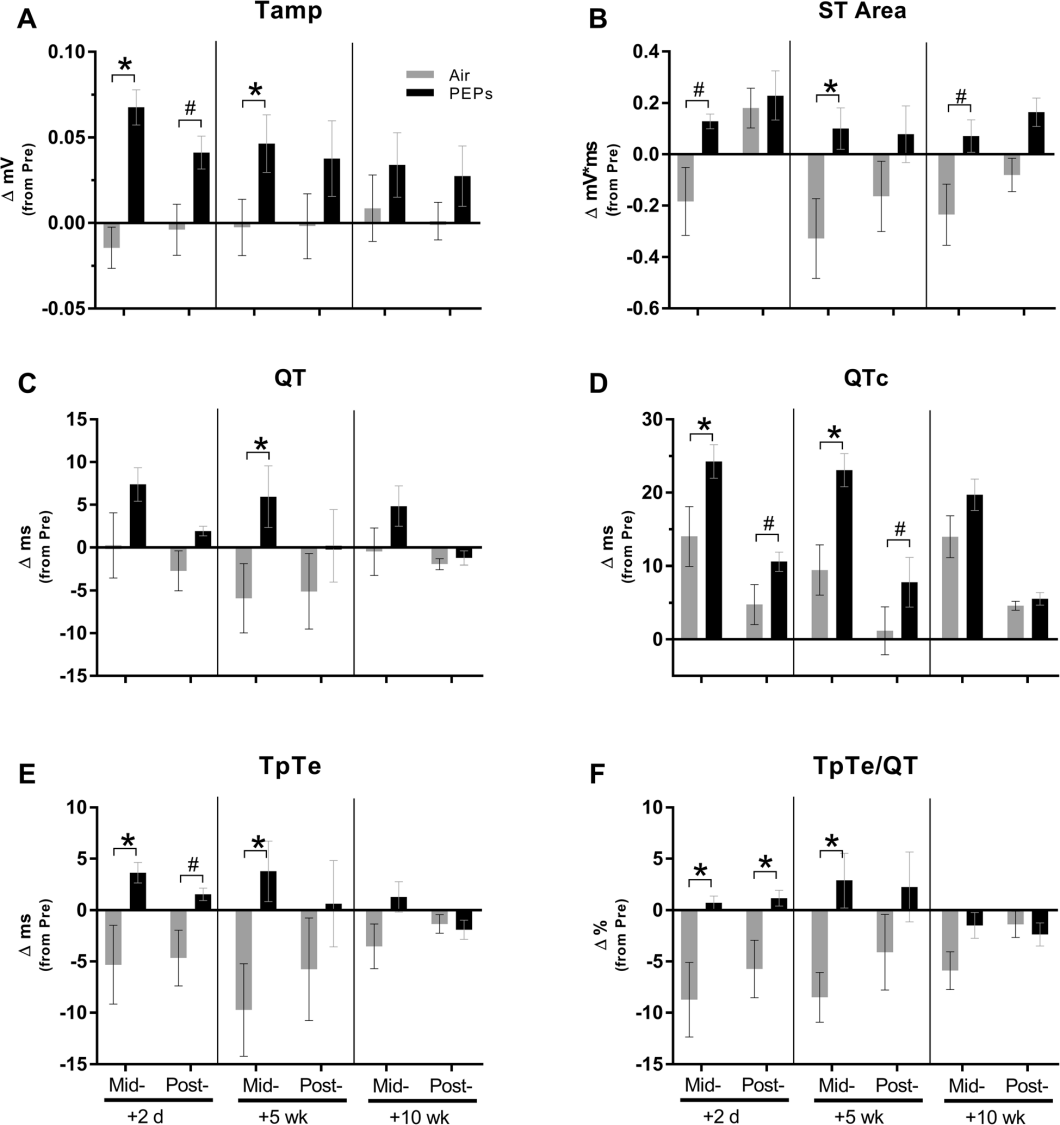
Changes in cardiac repolarization during and after 20-min stress tests at 2 days, 5 weeks, and 10 weeks after PEPs (**a**-**f**). Values are means ± standard errors of changes from 20-min pre-stress periods on 2, 35, and 70 days after cessation of inhalation exposures during Mid- and Post-Stress periods (20 min each) for each group, PEPs *n* = 4, Air *n* = 4. ^#^*P* < 0.10 and *P < 0.05 vs. Air. Group means of each animal’s average pre-stress values (across the three stress test days) ± SEM for Air and PEPs groups, respectively: Tamp = 0.060 ± 0.006 and 0.054 ± 0.007 mV; ST Area = − 0.822 ± 0.074 and − 0.807 ± 0.053 mV*ms; QT = 61.4 ± 4.1 and 56.9 ± 4.3 ms; QTc = 60.8 ± 4.2 and 56.4 ± 2.5 ms; TpTe = 32.6 ± 3.9 and 25.9 ± 2.1 ms; TpTe/QT = 0.51 ± 0.03 and 0.44 ± 0.01

Among Air rats, the stress test robustly increased HR (20-min mean ± SEM: 496 ± 2 BPM on day 2, 483 ± 8 BPM on week 5, and 468 ± 13 BPM on week 10). PEPs did not alter this response (Additional file 1: Figure S5). However, overall (across all stress days), PEPs increased *dP/dt*_max_ and decreased *dP/dt*_min_ during stress recovery while also accelerating RT and increasing developed pressure (devP; Table 2; all *P* < 0.05 vs. Air), collectively indicating PEPs enhanced hemodynamic responses to stress. Overall, PEPs prolonged all five indices of repolarization, including QTc, during the stress challenge or recovery (Table 2). Stress tests consistently increased VPBs relative to pre-stress among both groups, with no group differences in number of VPBs during stress (Additional file 1: Figure S6). Two days after the 21-day inhalation exposure, the stress test revealed a PEPs-induced decrease in EjeT absent of any other effects on LVP or HRV (Additional file 1: Table S3), and concomitant with increases in several measures of repolarization, including QTc and TpTe (Figs. 6d and 6e). Additionally, the ratio of TpTe to QT (an index of repolarization heterogeneity predictive of ventricular tachycardia and fibrillation [54]) remained increased during stress recovery (Fig. 6f; all *P* < 0.05 vs. Air). These effects of PEPs on repolarization were recapitulated during stress at 5 weeks after exposure (Fig. 6) and were followed during stress recovery by an increase in VPBs (Additional file 1: Figure S6; *P* = 0.05 vs. Air), *dP/dt*_max_, and devP, and a decrease in *dP/dt*_min_ (Additional file 1: Figure S5 and Table S3; all *P* < 0.05 vs. Air). At 10 weeks post-exposure, PEPs decreased *tau* both during and after stress (Additional file 1: Table S3; *P* < 0.05 vs. Air), indicating augmented diastolic function. PEPs did not significantly alter HRV during any of the stress test days.

#### Long-term effects on thermoregulation

Exposure to PEPs consistently increased resting core temperature (T_co_) by 0.4–0.5 °C (Additional file 1: Figure S7, *P* < 0.05 vs. Air). At 2 days post-exposure, stress decreased T_co_ in the PEPs group, restoring it to values comparable to the Air group. However, during the subsequent two stress tests, T_co_ remained elevated in PEPs rats relative to the Air group.

#### Biochemical effects after 10-week recovery

Urine samples were collected on the day before and the day after the final stress test (10 weeks post-PEPs) and assessed for changes in catecholamines and their metabolites to determine if PEPs persistently altered neurohormone excretion consistent with sympathetic dominance. In a preliminary assay, PEPs significantly increased urinary norepinephrine on the day after stress relative to the day before stress, whereas the Air-exposed group showed no such effect (Additional file 1: Figure S8). To validate these findings, we assessed a panel of biogenic amines using mass spectroscopy (Additional file 1: Figure S9). Across the two sampling days, PEPs caused an overall increase in dopamine (*P* = 0.05) as well as a trend of overall decreased metanephrine (*P* = 0.06). To determine if PEPs altered enzymatic metabolism, ratios of metabolites to parent compounds were compared between exposure groups [55, 56]. PEPs did not significantly affect indices of catechol-O-methyltransferase (COMT) metabolism (normetanephrine / norepinephrine, 3-methyltransferase / dopamine, and metanephrine / epinephrine), monoamine oxidase (MAO) metabolism (vanillylmandelic acid / metanephrine, vanillylmandelic acid / normetanephrine), or combined aldehyde dehydrogenase 2 and MAO-A metabolism (5-hydroxyindoleacetic acid / serotonin) at either pre-stress or post-stress (Additional file 1: Figure S10) [57], suggesting PEPs did not alter enzymatic metabolism despite changes in dopamine and metanephrine levels, but instead increased synthesis and/or secretion.

#### Cardiac protein expression after 10-week recovery

**Fig. 7:**
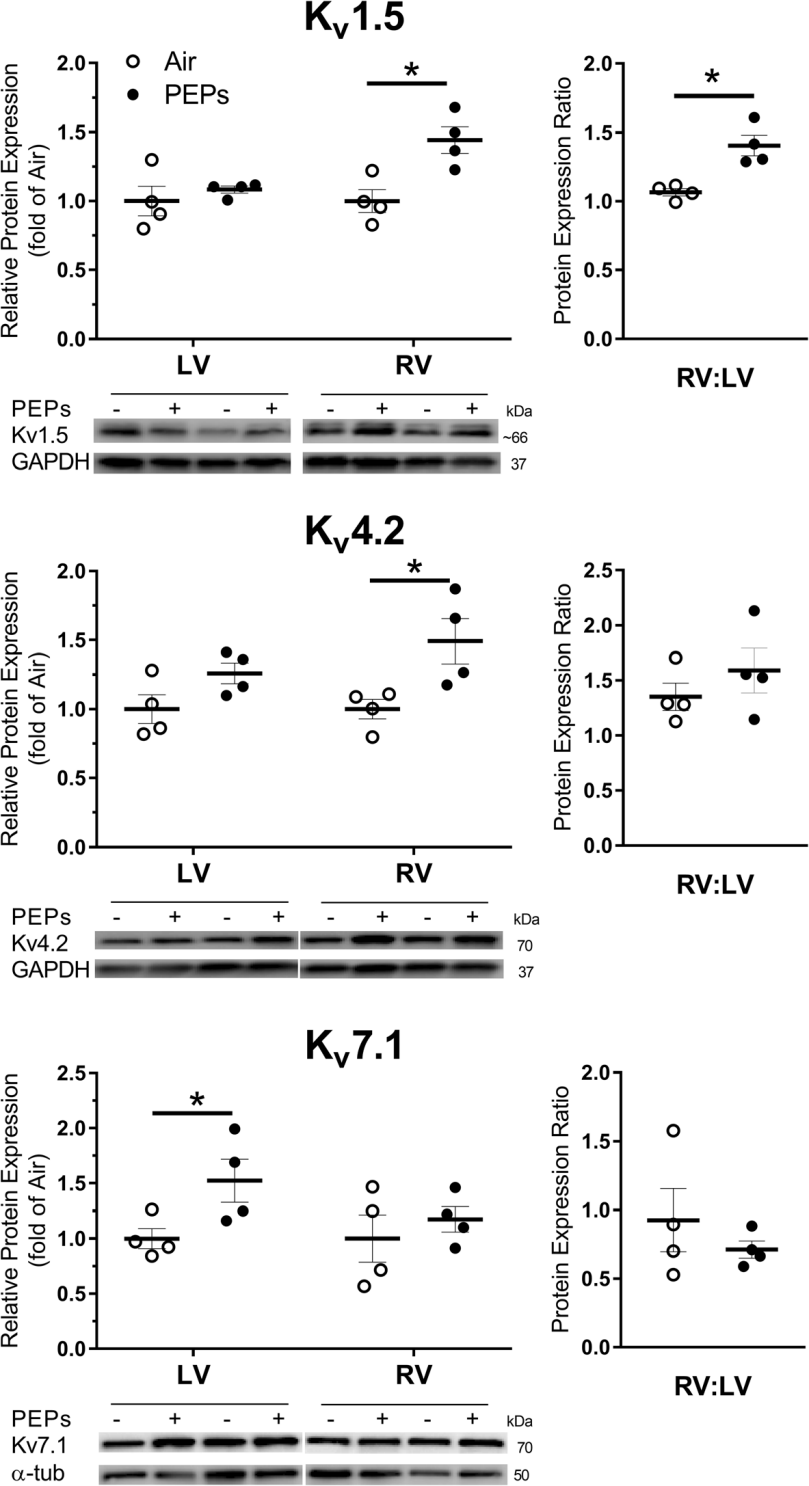
PEPs increases protein expression of repolarizing voltage-gated potassium channels in right and left ventricular myocardium**.** Horizontal bars indicate mean (± SEM) fold-difference from Air in expression, normalized to GAPDH. RV:LV represents the ratio of relative protein densities for GAPDH-normalized RV to LV. Individual animal values are indicated by open (Air) or closed (PEPs) circles

To determine whether known molecular mediators of ventricular repolarization defects and arrhythmia were affected by PEPs, we assessed protein expression of voltage-gated potassium channels key to ventricular repolarization (K_v_1.5, K_v_4.2, K_v_4.3, and K_v_7.1) at 10 weeks post-PEPs [58–60]. PEPs significantly increased LV K_v_7.1 (alias K_v_LQT1 or KCNQ1; Fig. 7). Importantly, β_1_-adrenergic receptors (β_1_ARs) expedite repolarization during sympathetic stimulation by phosphorylating K_v_7.1 protein at serine residues [61]. Because, even weeks after exposure, PEPs impaired repolarization during stress, and because this trait is pathognomonic of concealed Long QT Syndrome 1 (LQT1) [62, 63] which involves mutations in —or impaired phosphorylative regulation of —K_v_7.1, we assessed K_v_7.1 serine phosphorylation through immunoprecipitation but found no differences at 10 weeks post-exposure (Additional file 1: Figure S11). PEPs also significantly increased K_v_1.5 and K_v_4.2 expression in the RV (Fig. 7; *P* < 0.05 vs. Air) without affecting LV expression, leading to a 32% greater RV:LV ratio for K_v_1.5 relative to the Air group (*P* < 0.05), which expressed K_v_1.5 equally between the ventricles. PEPs did not affect K_v_4.3 expression in either ventricle (data not shown). We also assessed phosphorylation of ERK1/2 (which inversely regulates Kv1.5 expression [64] and is stimulated by adrenergic receptor activation) and expression of β_1_ARs (which is central to sympathetic enhancements in cardiac conduction, ventricular performance [65], and repolarization [61]) but found no significant effects of PEPs (Additional file 1: Figures S12 and S13).

## Discussion

Exposure to PEPs at occupationally relevant levels [30, 66] altered cardiac function, autonomic regulation, and expression of essential repolarizing ion channels. Effects included increases in LV systolic pressure, QT interval, ventricular tachyarrhythmia, and sympathetic influence, along with declines in measures of contractility and trends of decreased baroreflex sensitivity (Additional file 1: Table S5). In addition, PEPs induced cardiac electrical instability, characterized by P wave shortening during and after exposures, QT prolongation immediately after exposures, and spontaneous ventricular arrhythmias and stress-evoked QT prolongation at up to 5 weeks after exposures. Even at 10 weeks after exposure, PEPs induced basal systolic hypertension, decreased EjeT, and increased renal dopamine excretion concomitant with increased ventricular expression of repolarizing channels (K_v_7.1, K_v_1.5, and K_v_4.2). It is worth noting that most of the observed effects are individually associated with cardiovascular morbidity and mortality and together may synergistically increase cardiac risk [36, 47, 67, 68], particularly via hemodynamic stress, heart failure decompensation, atherogenesis, plaque rupture, thrombosis, and/or arrhythmia. Thus, our observations accord with evidence that short-term PM exposures promote CVD and specifically implicate autonomic imbalance and repolarization defects in adverse cardiac outcomes.

PEPs induced sympathetic dominance and decreased contractility during exposure. Unlike LV *dP/dt*_max_, which fluctuates with afterload (aortic pressure) and preload (venous return) [35], CtrI provides a stable, load-independent in vivo marker of systolic cardiac performance [35]. Decreases in EjeT can further signify decreased contractility [37] and predict heart failure [36]. Thus, declines in both EjeT and CtrI suggested that PEPs exposure may impair contractility despite HRV indicating enhanced sympathetic influence. These findings accord with the negative inotropic effects of exposures at > 2-fold higher PM concentrations of carbon black [33, 34], diesel exhaust [31], or concentrated ambient PM [69], which may decrease cardiac output while inducing catecholamine surges to provoke hypertension, pulmonary edema, and intracellular signaling toward cardiac remodeling [65]. Accordingly, PEPs impaired contractility indices *during* exposure especially on day 21, but these effects rapidly dissipated *immediately after* exposure when systolic pressure increased—potentially through neurohormonal compensation. Likewise, systolic pressure remained increased up to 10 weeks thereafter, when dopamine—the parent catecholamine of norepinephrine and epinephrine—was also increased.

Hypertension is the leading cause of hypertensive heart disease (including heart failure, ischemic heart disease, and LV hypertrophy) and cardiovascular mortality [1]. Even subtle increases in systolic pressure that do not qualify as clinical hypertension may cause over one third of cardiovascular deaths [70]. PM exposure promotes hypertension, heart failure, and atherosclerosis [3]. We speculate from our findings that exposure to PEPs—and perhaps PM in general— precipitates and exacerbates hypertensive heart disease by jointly impairing contractility and increasing arterial pressure. The time-course of effects indicates a mode of action underlying PM-induced heart failure exacerbation, as systolic dysfunction initiates compensatory neurohormonal and hemodynamic changes that promote pulmonary edema and decompensation. However, given the low animal numbers in this study, these findings remain relatively preliminary. More direct assessments of LV performance and pulmonary edema, and use of larger experimental groups, susceptible animal models, molecular interventions, and long-term exposures, are needed to validate our observations and further elucidate the underlying mechanisms.

The final day of PEPs exposure was accompanied by sympathetic dominance, followed immediately after exposure by increased systolic pressure (+ 18 mmHg) and accelerated EMC. This slight delay between sympathetic dominance and hypertension suggest the hemodynamic effects stemmed partly from autonomic imbalance. PEPs also increased systolic pressure even at 10 weeks post-exposure concomitant with increased dopamine excretion. Because enzymatic activity did not appear significantly altered, PEPs likely increased catecholamine release and/or synthesis. Although unclear from our measures, surges in dopamine immediately after exposure may have enabled the rapid recovery of contractility, increase in systolic pressure, and acceleration of EMC absent of any concurrent HRV effects, as dopamine increases contractility and systolic pressure disproportionate to its effects on pacemaker activity [71–73]. A few studies have suggested links between PM and dopamine, including one that tied “falling dust” to increased urinary dopamine and its metabolite, norepinephrine [74]. In recent human studies, annual PM_2.5_ levels were associated with increasing urinary dopamine and epinephrine [75], and a 9-day PM_2.5_ exposure corresponded with increases in systolic blood pressure and circulating tyrosine (dopamine’s parent compound) and norepinephrine [76]. Dopamine and norepinephrine alter cardiovascular physiology via stimulation of dopaminergic and adrenergic receptors, and can chronically desensitize and/or downregulate these receptors via G-protein receptor kinases (GRKs) and hyper-phosphorylation [65, 77, 78]. Accordingly, PM can induce hypertension through GRK-mediated desensitization and downregulation of renal D1 dopamine receptors [79], but the role of elevated dopamine remains unknown. Moreover, as β_3_ adrenergic and D1 dopamine receptors mediate thermogenesis [65, 77], catecholamine elevations may also account for the body temperature increases over the entire 10 weeks following PEPs exposure. Ultimately, with more prolonged inhalation exposures, chronic sympathetic activation may provoke cardiac structural and metabolic remodeling toward persistent declines in cardiac performance [65], as previously demonstrated [32]. Together, the effects of PEPs on HRV and catecholamines, concomitant with hypertension, contractility decrements, arrhythmia, and repolarization defects, add further evidence that exposure to PM increases risk for arrhythmia, heart failure exacerbation, and reperfusion injury, through sympathetic predominance [31, 80–84]. Moreover, our observations suggest that the adverse outcomes associated with ambient PM may translate to pulmonary exposures to ENMs.

To further elucidate the etiology of PEPs-induced hypertension, we assessed BRS on the two exposure days with greatest pressure effects (days 9 and 21). Baroreflexes provide a homeostatic defense against pressure overload during pressure increases by provoking parasympathetic dominance to slow heart rate. Spontaneous BRS tended to decrease equally *during* each of the two PEPs exposures and rebounded immediately afterwards. Although pressure remained unaffected *during* exposure, PEPs increased systolic pressure *after* each exposure. The concomitant pressure increase with a restoration of BRS slope suggests PEPs restored BRS at a higher mean systolic pressure through ‘baroreflex resetting’ [85]. We previously found in rats with metabolic syndrome that traffic PM simultaneously decreased BRS and HRV, with equivalent effects on BRS (− 0.3 ms/mmHg) and similar correlations between BRS and HRV as found here [86]. Thus, similar to other PM, PEPs exposure likely promotes hypertension not only through autonomic imbalance but also via impaired baroreflexes.

Exposure to PEPs prolonged QT, altered expression of key repolarizing potassium channels in the right ventricle (K_v_1.5, K_v_4.2) and left ventricle (K_v_7.1), and increased arrhythmia long after cessation of exposures, indicating PEPs induced spontaneous tachyarrhythmia and electrical remodeling. At post-exposure on every day analyzed for ECG morphology (days 1, 9, and 21), PEPs prolonged QTc overall, recapitulating prior clinical and toxicological observations of the cardiac effects of PM exposure [38–45]. Increases in QT and TpTe (a subcomponent of QT) are associated with LV remodeling and can precipitate severe arrhythmia and cardiac mortality [47, 48, 87]. Likewise, PEPs increased spontaneous ventricular premature beats at both 2 days and 5 weeks after the 21-day exposure, complementing epidemiologic associations between PM exposure and spontaneous ventricular arrhythmia [81, 88–91] and sudden cardiac arrest [92, 93]. Interestingly, aerosol exposures in noninvasive rat models of CVD typically provoke spontaneous atrioventricular block arrhythmias [45, 86, 94–98], which differ from the premature ventricular ectopy that predominates with PM exposure in humans. Yet, rodent models of surgical myocardial infarction [99–101] or genetic dilated fibrotic cardiomyopathy [102] have more consistently demonstrated tachyarrhythmias with PM exposures. Chronic LV catheterization surgery in our current study may have increased susceptibility to ventricular arrhythmia, as it leaves a small fibrotic apical scar (< 2 mm diameter). LV scarring can impair conduction, propagate ectopy, and prolong QT and TpTe [103]; however, repolarization at baseline was no different from telemetered rats naïve of thoracic surgery. Meanwhile, PEPs impeded acceleration of repolarization during increased heart rate (i.e., repolarization reserve), manifesting as prolonged QTc, TpTe, and TpTe/QT during stress up through 5 weeks after exposure. To prevent arrhythmia during sympathetic-induced increases in heart rate, healthy cardiomyocytes (rat and human) accelerate repolarization by enhancing the IK_s_ current [104] via K_v_7.1 phosphorylation [61]. Importantly, the long-term physiologic effects of PEPs resembled patients with concealed Long QT (LQT) Syndrome 1, who have a mutation in K_v_7.1 (a major phosphorylation target of β_1_AR [61]) and prolonged QT and TpTe only during sympatho-excitation [62, 63]. Due to an inability to decrease QT proportional to RR, patients with LQT1 have a high risk of fatal arrhythmia, especially during sympathetic activation with physical exertion or stress [105]. Although stress tests revealed QT prolongation in PEPs-exposed rats, this effect was insufficient to evoke arrhythmia. Effects on QTc, TpTe, and arrhythmia abated by 10 weeks post-exposure, when PEPs increased expression of LV K_v_7.1, and RV K_v_1.5 (I_Kur_ current) and K_v_4.2 (I_to_ current) by roughly 50%, and significantly altered the balance of K_v_1.5 expression between LV and RV, which may increase repolarization heterogeneity and arrhythmia susceptibility. Yet, QT prolongation and spontaneous arrhythmia have also been attributed to 30% decreases in ventricular K_v_1.5 protein expression in mice [64]. Thus, the QT-prolonging and arrhythmogenic effects of PEPs may have subsided by week 10 post-exposure due to compensatory enhancements in K_v_ expression. Conversely, increased K_v_1.5 expression can increase excitability and spontaneous activation in rat cardiomyocytes [59], and increased K_v_1.5 in one ventricle but not the other may increase myocardial excitability by increasing dispersion and asynchronous repolarization between LV and RV [65, 106, 107]. Thus, our electrophysiological and molecular observations suggest PEPs exposure promotes cardiac arrhythmia through electrical remodeling. These findings warrant follow-up studies to quantify the prevalence and incidence of arrhythmia in printshop workers and assess the electrophysiologic effects of occupational exposures to laser printer aerosols.

*Tau* and RT are inverse indices of diastolic function that reflect relaxation properties of the myocardium, albeit less reliably than CtrI indicates contractile properties [35]. *Tau* positively correlated with HRV in PEPs rats, suggesting exposure-induced sympatho-excitation coincided with a positive lusitropic effect. The changes in *tau* and RT contrast with implications of impaired systolic function (decreased EjeT and CtrI) with PEPs exposure. Nonetheless, systolic and diastolic dysfunction often involve distinct molecular mediators and may occur independent of each other or in compensatory opposition of the other. Notably as well, PEPs eroded associations between HRV and CtrI, and thus may have disrupted normal autonomic modulation of inotropy. Indeed, inotropy and lusitropy can be divergently affected by protein kinase C phosphorylation of cardiac troponin I upon stimulation of either adrenergic receptors or non-autonomic receptors (angiotensin or endothelin) [108, 109]. Months after the negative inotropic effects of exposure, we did not see any effects on ventricular troponin I phosphorylation, but the relevance of these observations to preceding physiologic effects is unclear. Both elevations in sympathetic influence and declines in LV systolic function during PEPs exposure suggest pathogenic effects that may acutely and transiently exacerbate underlying heart failure.

Several limitations of this study merit discretion when interpreting our findings. Firstly, this study was designed to delineate gross effects of PEPs exposures on cardiac mechanical function and related measures. Yet, due to the demanding LV catheterization surgeries and a limited number of telemeters, the control and treatment groups were likely underpowered (*n* = 4/group) for several endpoints. Because one Air rat had recurrent LV pressure artifacts during exposure, we excluded it from analyses at this phase of the study. Because its waveform morphology normalized after the 21-day PEPs exposure but absolute pressure values remained invalid (e.g., LVEDP = − 40 mmHg), LVP endpoints sensitive to absolute pressure values were excluded for this rat but time intervals (e.g., RT and EjeT) were included. The limited number of animals in this study, particularly for the pressure-derived parameters with Air *n* = 3 during exposures, increases likelihood of Type II, and to a lesser extent Type I, errors. Yet, to optimize group comparisons, each animal’s physiologic response to treatment was normalized according to its own four-day baseline. Additionally, on monitoring days after the 21-day PEPs regimen, animals were placed in ambulatory cages with bedding, food, and room to forage, potentially obscuring any effects on LVP and HRV on post-exposure days. Separately, while our findings implicate electrical remodeling and sympathetic dominance in PM-induced arrhythmia and hypertension, this study was not designed to definitively confirm these as the underlying mechanisms. More mechanistic studies are need to fully understand the potential multiple mechanisms implicated with the observed endpoints. Finally, while PEPs was associated with decreased EjeT and trends of decreased CtrI, additional measures of LV mechanical performance (e.g., pressure-volume relationships) could provide more definitive evidence toward LV systolic dysfunction; however, such endpoints require anesthetics that alter cardiac function and may thus mask treatment-related effects [110]. Ultimately, the confluence of our observations across multiple endpoints and repeated days consistently suggests adverse effects of PEPs on cardiac rhythmicity, autonomic balance, mechanical performance, and systolic arterial pressure.

As noted, the exposure concentrations of PEPs in this study are within the ranges of observed concentrations at commercial photocopy centers [21, 30]. Photocopiers and laser printers use near-identical processes with nano-enabled toner formulations, and thus generate similar aerosols containing engineered nanomaterials, VOCs, PAHs, and transition metals, in addition to organic carbon particulates [21]. Importantly, transition metals and PAHs have been implicated in the adverse cardiopulmonary and autonomic effects of PM [111–113], including increased expression of K_v_1.5, K_v_4.2, and K_v_7.1 (K_v_LQT1) in cardiac myocytes exposed to Zn PM [114]. Additionally, there is growing appreciation that ultrafine particles (aerodynamic diameter < 100 nm), similar in size to PEPs, bear profound cardiovascular toxicity per-mass when compared to accumulation mode PM (PM_0.1–2.5_) because of higher surface area per unit mass, and enhanced alveolar deposition and systemic translocation for direct interactions with cardiovascular cells [15]. Prior occupational studies have reported associations between PEPs exposures and oxidative stress, DNA damage, systemic inflammation, respiratory infection, and diminished pulmonary function [21]. Likewise, it is worth noting that in our recently published companion paper on the effects of PEPs on lung injury and inflammation, elevated levels of inflammation and oxidative stress markers were found in the blood of the exposed animals [53]. To our knowledge, this study is the first to report on the cardiac effects of exposure to PEPs, and in so doing, hails a novel health risk of a modern and ubiquitous technology.

## Conclusions

The magnitude and breadth of the current study’s findings, along with the lack of exposure control technologies in commercial printing facilities [66], bear stark implications for photocopy center employees with underlying CVD or related susceptibilities, including pregnancy. We are unaware of any current policies or regulations at local, state, or federal levels that pertain to laser-based printer or copier emissions. Our findings, with validation from further studies, may compel regulatory agencies and industry to establish occupational exposure limits and apply technological safeguards so as to decrease the levels and toxicity of printer aerosol exposures. This investigation yielded novel evidence that exposure to engineered nanomaterials released across the life-cycle of a nano-enabled product increases cardiovascular risk. Moreover, the findings represent an important advancement in inhalation toxicology, as they offer a seminal demonstration of the real-time effects of inhaled PM on LV performance in rodents. Overall, repeated exposure to PEPs impaired both cardiac mechanical performance and repolarization, and increased arterial pressure and ventricular arrhythmia. PEPs also altered multiple cardioregulatory components of the autonomic nervous system consistent with sympathetic activation, including decreased HRV, increased body temperature, and increased catecholamine production. These observations complement the mounting evidence that autonomic dysregulation mediates the pathophysiologic effects of inhaled aerosols on cardiac function. Collectively, these data demonstrate that printer emitted aerosols, and perhaps aerosols from nano-enabled products in general, present significant health risks through adverse effects on the cardiovascular system, with key implications for health risks in occupational settings.

## Methods

### Whole-body inhalation exposure to PEPs

Rats were housed in individual whole-body exposure chambers as previously detailed by the authors [27, 53]. The exposed group of rats housed in individual chambers received PEPs and gaseous pollutants emitted by a laser printer B1 using the Printer Exposure Generation System (PEGS) as previously detailed by the authors [27] and described further in a recently published companion study [53]. Printer B1 was selected to generate PEPs emissions by printing a 5%-page coverage monochrome document using standardized settings [27]. In parallel, another group of rats was exposed to High Efficiency Particulate Air (HEPA)-filtered air. An empty exposure chamber was sampled continuously throughout the study for aerosol characterization.

### Real time measurements of PEPs and other environmental conditions throughout exposure

Particle number concentration, size distribution, temperature, relative humidity, and total volatile organic compounds (tVOC) levels were measured in real time in one of the twelve animal inhalation exposure chambers throughout the exposure durations. A scanning mobility particle sizer (SMPS Model 3080, TSI Inc., Shoreview, MN) was also used for measuring the particle number concentration and size distribution (ranging from 2.5 to 210 nm) in the chamber. Real-time tVOCs levels were also monitored using a tVOC monitor (Graywolf Sensing Solutions, Shelton, CT). All the real time instruments were calibrated, and background tests were performed at the beginning of each sampling experiment. No significant variation in the temperature (°C) and relative humidity (%) in the inhalation animal chambers was observed throughout the exposure period.

### Animals and surgery for telemeter implantation

Animals were housed and treated in accordance with the National Institute of Health guidelines for the care and use of laboratory animals. All animal protocols were approved by the Harvard Medical Area Institutional Animal Care and Use Committee (IACUC), with the surgical procedures also approved by the IACUC of DataSciences International (DSI, St. Paul, MN). Eight male Sprague Dawley rats (225–245 g, 52 days old, Charles River Laboratories, Kingston, NY) were implanted with radiotelemeters (HD-S21) capable of measuring ECG and two pressure signals. Surgeries were performed by trained surgeons at DSI (see Supplement for details). Animals were allowed 20 days to recover from surgery, after which LVP and ECG waveforms were confirmed as stable. Rats were then shipped to Harvard, where they received standard chow (irradiated PicoLab Rodent Diet 205,053, Lab Diet, St. Louis, MO) and water ad libitum in standard polycarbonate 17.6-L rat cages over a 12-h light/dark cycle. After a three-week quarantine in the animal facility, rats were acclimated to HEPA-filtered room air delivered at 1.5 L/min in 1.4 L-whole-body exposure chambers in a previously-described exposure system [115] for 1 h in our Inhalation Toxicology Laboratory at HSPH while acquiring radiotelemetry signals.

On each of the following 4 days, rats were exposed 6 h to HEPA-filtered Air for BL data. Mean BL heart rate and maximal pressure upslope (*dP/dt*_max_, a rough measure of contractility), were graphed on an X-Y plot and pairs of proximal rats were evenly divided among the Air-control or PEPs groups (*n* = 4 / group). On the next day, animals were placed in exposure chambers and monitored by telemetry for 6 h, involving 30-min pre-exposure, 5-h exposure, and 30-min post-exposure periods, each day for 21 continuous days. Data were analyzed on all BL exposure days, days 1, 5, 9, 13, 17, 20, and 21 of exposure, and all stress test days (Fig. 2). One day after the final exposure, animals were placed in ambulatory monitoring cages (standard polycarbonate mouse cages, 25 × 16 × 13 cm, or 5.2 L) with bedding and limited chow (two pieces) and monitored on the exposure table at the same time of day for the same duration as the prior exposures, including pre- and post-exposure phases (6 h). These cages allowed for freedom of movement, including squatting on hind-limbs.

#### ECG and LVP

ECG and LVP waveforms were analyzed for multiple endpoints on select exposure days, and treatment-related differences were determined by comparing time-matched changes from BL (the average of four sham exposures) between groups. In addition to analyses on BL days, HRV and LVP parameters were analyzed at four-day intervals and the penultimate day (exposures 1, 5, 9, 13, 17, 20, and 21); ECG morphology was analyzed on exposure days 1, 9, and 21; BRS was analyzed on exposure days 9 and 21; and electro-mechanical coupling (EMC, time from ECG Q to LV EDP, Additional file 1: Figure S15) was analyzed on exposure days 1, 9, 20, and 21. All HRV and LVP parameters were assessed over the sham exposure immediately following day 21.

ECG waveforms were analyzed with ecgAuto, v3.3 (Emka Technologies, Paris, France) for mean RR intervals, HRV, and arrhythmia as we have previously described [86, 94]. A library of 224 manually marked representative PQRST complexes was used to identify beat landmarks for ECG analyses according to previously described criteria [86]. ECG analyses were performed on all 4 BL days, inhalation exposure days 1, 9, and 21, and all three stress test days. On exposure days, T_end_ was marked inaccurately by the software such that extensive manual correction was required to include T_end_-derived measures (e.g., Q-T_end_, TpTe, etc.) in any assessments of exposure-related effects. We thus defined QT on the three exposure days (6 h each) as Q-T_peak_, which was analyzed in conjunction with Q-T_end_ on BL days. Conversely, because stress-test days were brief (only 1 h of continuous waveforms) and thus facilitated rigorous inspection and manual correction of all T_end_ markings, QT was defined as Q-T_end_ unless otherwise indicated (Table 2). Per our more rigorous validation of repolarization markings on stress days, we also assessed change in repolarization-related ECG parameters on stress days based on change from the 20-min pre-stress phase. QTc was calculated by the previously described murine-specific formula [116], which we adapted for rats as QT÷(RR/190)^1/2^ based on the average RR (190 ms) in the Air group across all analyzed exposure days.

We excluded arrhythmias (> 18% reduction or > 25% increase in RR relative to the average of the prior 4 RRs) and, on select days, identified and quantified them as previously described [86] while blind to treatment and with verification by examination of concurrent LVP waveforms. ECG waveforms were analyzed in 5-min segments continuously over all BL days, select exposure days (1, 5, 9, 13, 17, 20, 21), a recovery day (day 22), and all stress test days. HRV analyses generated the time-domain variables SDNN and RMSSD, as well as the frequency-domain variables, HF (0.75—3.50 Hz), LF (0.20—0.75 Hz), and their ratio (LF/HF) as previously described [86, 94].

LVP signals were filtered of malformed waves, removing irregularly shaped contraction cycles as described [117], but with delineation in ecgAuto by removing all waveforms below 0.1% or above 99.9% the frequency distribution for LVEDP, LVESP, *dP/dt*_max_, CtrI (*dP/dt*_max_ normalized by concurrent pressure to control for afterload), peak downslope in pressure (*dP/dt*_min_), and the lusitropic index, *tau* (the time required for *dP/dt*_min_ to reach half its value). Subsequently, any beats appearing as outliers in scatterplots of these parameters as well as software-defined begin diastolic pressure (BDP), begin systolic pressure (BSP), and maximum systolic pressure (maxSP), were visually inspected and removed if misshaped. To more thoroughly filter waveform distortions to ensure accurate slopes, we derived *dP/dt* parameters after removal of beats with LVESP and BSP diverging by > 15 mmHg. All other pressure parameters were derived after additional removal of beats with EDP < − 1 mmHg to correct for rare instances of signal drift. CtrI is a particularly reliable afterload-independent marker of mechanical performance [35]. RT and *tau* reflect changes in lusitropy (diastolic function), albeit modestly, whereas LV *dP/dt*_max_ and *dP/dt*_min_ are prohibitively sensitive to changes in afterload and preload [35]. Cycle lengths were assessed for ejection time (BSP-ESP interval) and relaxation time (ESP-EDP interval). One rat in the Air group was excluded from LVP endpoints on BL and inhalation exposure days due to recurrent abnormalities in pressure waveform morphology and amplitude. Because waveform morphology normalized after cessation of the PEPs exposure regimen but pressure amplitudes remained inconsistent, on stress test days this rat was excluded only from endpoints sensitive to pressure amplitude.

### Stress tests

To further unmask any latent effects of PEPs on cardiac electrophysiology, we assessed ECG morphology and arrhythmia during and after a cold-water stress test. On stress test days (2, 27, and 70 days after final PEPs exposure day), telemetry signals were acquired from rats in their home cages for 20 min. Thereafter, animals were transferred to a rat cage with 2 cm deep ice-free chilled water (1–3 °C maintained throughout stress) for 20 min, and returned to their home cages for another 20 min [52]. Except where indicated otherwise (i.e., VPBs and select repolarization calculations), ECG and LVP parameters were normalized by the mean of BL days preceding inhalation exposure.

### Tissue collection

On the days before and after the final stress test (10 weeks post-PEPs), rats were placed in metabolic chambers between 1000 and 1300 and urine collected and frozen immediately. Telemetered rats were euthanized and necropsied 2 days after the 10-week post-exposure stress test. Animals were fully anesthetized by 3% isoflurane and exsanguinated with blood collection from the abdominal aorta. Blood was collected in K_2_-EDTA-buffered collection tubes and spun at 3000 RPM, and plasma aliquots were collected and frozen at − 80 °C immediately thereafter. Hearts were rapidly excised, placed on ice, rinsed free of blood with cooled saline, trimmed free of fat, longitudinally sectioned along the atrial axis, and one section placed in formalin fixative. For the remaining section, the RV was dissected from the LV and interventricular septum, and all tissues were placed in cryovials, snap-frozen in liquid nitrogen, and stored at − 80 °C immediately thereafter.

### Analyses of urine, blood, and heart samples

Urine was analyzed by an enzyme-linked immunosorbent assay (ELISA) kit for norepinephrine (Eagle Biosciences, NOU39-K010) and then analyzed for biogenic monoamines, including catecholamines and serotonin, using ultrahigh performance liquid chromatography with tandem mass spectrometry (UPLC-MS/MS) in the Metabolomics Core of the University of Louisville’s Diabetes and Obesity Center as previously described [57]. For UPLC-MS/MS analysis of dopamine, norepinephrine (NE), epinephrine (EP), serotonin (5-HT), and their metabolites (metanephrine [MN], normetanephrine [NMN], vanillylmandelic acid [VMA], 3-methoxytyramine [3-MT], and 5-hydroxyindole-3-acetic acid [5-HIAA]), urine samples were thawed on ice, vortexed and diluted 1:50 with 0.2% formic acid containing isotopic labeled internal standards. 1 μL of mixture was analyzed on an UPLC-MS/MS instrument (ACQUITY UPLC H-Class system and Xevo TQ-S micro triple quadrupole mass spectrometer, all from Waters Inc., MA). Separation was performed on an Acquity UPLC HSS PFP (150 mm × 2.1 mm, 1.8 μm) column (Waters Inc., MA) with a binary gradient comprised of 0.2% formic acid (Solvent A) and methanol (Solvent B). Three multiple reaction monitoring (MRM) transitions were set up for each sample: one for quantification, one for confirmation, and one for labeled internal standard. At least 12 data points were collected for each peak. Analytes were quantified using peak area ratio based on 8 point-standard curves run before and after the urine samples, and analyte concentrations were normalized by creatinine measured on a COBAS MIRA-plus analyzer (Roche, NJ) with Infinity Creatinine Reagent (Thermo Fisher Scientific, MA). Blood was assayed for B-type natriuretic peptide (BNP-45, AssayPro ERB1202–1), n-terminal propeptide of atrial natriuretic peptide (NT-proANP, Biomedica BI-20892), and cardiac troponin I (Life Diagnostics, CTNI-2-US Ultra-Sensitive Rat Cardiac Troponin-I) according to manufacturer-specified protocols. In a separate study, hearts from rats with chronic LVP implants identical to this study and exposed to filtered air in the same exposure system for 23 days were assessed for histopathological indications of hypertrophy and fibrosis relative to surgically-naïve control rats. Using Masson’s trichrome we found that the chronic apical catheterization resulted in a small fibrotic lesion (< 2 mm diameter), but relative to surgically naïve rats there were no significant effects on cardiomyocyte area (mean ± SEM: 510 ± 28 μm^2^ vs. 488 + 24 μm^2^, *P* = 0.45) or interstitial fibrosis (1.5 ± 0.1% vs. 1.1 ± 0.1%, *P* = 0.06) in the apical half of the LV, and no effects on the RV or the base of the heart, at 6–10 weeks post-surgery (unpublished data).

Right and left ventricles were separately homogenized in 1X RIPA buffer and immunoblot samples were made using Laemmli buffer with (or without) DTT (ThermoFisher). Equal amounts of protein (10–30 μg) were first separated via agarose gel electrophoresis (7, 10%, or AnyKd, BioRad) and then transferred wet to 0.2 μm PVDF or nitrocellulose (BioRad, GE Healthcare) membranes. Membranes were blocked 1 h at RT with 5% NFDM (Lab Scientific) before incubating overnight with primary antibody (1:250–1:5000 dilution, Cell Signaling [t-cTnI #4002; p-cTnI #4004; t-ERK #9102; p-ERK # 9101; t-Akt # 9272; p-Akt # 13038; GAPDH #2118), Abcam [Cxn43/GJA1 # ab11370; HO-1 #ab13243; Myostatin/anti-GDF8 #ab98337], Alamone [Kv1.5 #APC-004; Kv4.2 #APC-023; Kv4.3 #APC-017; Kv7.1 #APC-022], Santa Cruz [β1-AR #sc-568]) in either 5% BSA (Sigma) or 5% NFDM per manufacturer recommendation. Membranes were washed in TBST and then incubated 1 h at RT in 1:2000 secondary antibody (anti-rabbit with HRP, Cell Signaling) in 5% NFDM, then membranes were washed 3 × 15 min prior to development and imaging. Membranes were developed in ECL (ThermoFisher), dried, and imaged in real-time using MyImager (ThermoFisher) according to manufacturer’s protocol. All images were quantified in ImageJ, normalized to loading control. For Kv7.1 IP methods, see *Supplement.*

### Statistics

We analyzed time-series deltas (each animal’s change during exposure from the average of its four BL days) with linear mixed effects models (PROC MIXED) for day-specific or overall inhalant effects while controlling for day and selecting a random effects structure using AIC best fit criteria. Given their non-normal and longitudinal traits, we analyzed arrhythmia counts via generalized estimating equation (PROC GENMOD) as number of events per hour, assuming a Poisson distribution and exchangeable correlation structure. PROC REG was used to compare physiologic parameters (simple linear regression). Biochemical and molecular data were analyzed for group differences by two-tailed Student’s t-test except for biogenic amines, for which we performed repeated measures two-way analysis of variance with Sidak multiple comparisons test and Grubbs test for outliers, which were excluded from this analysis. Statistical significance was assumed at *P* < 0.05 between PEPs and Air control groups.

## Supplementary information

**Additional file 1: Results.** **Table S1.** Baseline characteristics of treatment groups. **Table S2**. Daily effects of PEPs exposure on LV pressure, HRV, and ECG morphology. **Table S3.** Daily effects of PEPs on LV pressure, HRV, and ECG morphology at 2 days, 35 days, and 70 days after exposure cessation, and before, during, and after individual 20-min stress tests. **Table S4.** Plasma ELISAs results. **Table S5.** Summary of physiologic effects of PEPs on cardiac hemodynamic, autonomic, and electrophysiologic function. **Figure S1.** Aerosol characterization over the 21-day exposure to laser printer-emitted particles (PEPs). **Figure S2.** Effects of PEPs on high frequency HRV during and immediately after exposure. **Figure S3.** Effects of PEPs on maximum LV pressure upslope (dP/dt_max_ ), minimum downslope (dP/dt_min_ ), and rate-pressure product. **Figure S4.** BRS slope during inhalation exposures. **Figure S5.** Changes in heart rate and LV pressure before, during, and after 20-min ice water stress tests at 2 days, 5 weeks, and 10 weeks after PEPs. **Figure S6.** Influence of PEPs on stress-induced ventricular premature beats. **Figure S7.** Effects of PEPs exposure and ice water stress on core body temperature before, during, and after ice water stress tests. **Figure S8.** Urinary norepinephrine analyzed by ELISA. **Figure S9.** Urine catecholamines at 10 weeks after cessation of PEPs exposure measured by HPLC MS/MS. **Figure S10.** Ratios of parent compounds to daughter metabolites for assessment of metabolic activity at 10 weeks after cessation of PEPs exposure. **Figure S11.** Serine phosphorylation of K_v_7.1 was not significantly altered by PEPs. **Figure S12.** PEPs does not significantly affect ventricular phosphorylation of ERK or AKT. **Figure S13.** Effects of PEPs on β_1_AR expression in the right and left ventricular myocardium. **Figure S14.** PEPs did not significantly affect RV cardiac troponin I (cTnI) phosphorylation or total heme-oxygenase 1 (HO-1) expression. **Figure S15.** ECG and LVP analysis. **Methods**.

## Data Availability

Data supporting the findings are found within the manuscript and supplemental material. Raw data files will be provided by the corresponding author upon request.

## Abbreviations

3-MT: 3-methoxytyramine
5-HIAA: 5-hydroxyindole-3-acetic acid
5-HT: serotonin
BDP: begin diastolic pressure
BNP: B-type natriuretic peptide
BPM: beats per minute
BRS: baroreflex sensitivity
BSP: begin systolic pressure
COMT: catechol-O-methyltransferase
cTnl: cardiac troponin I
CtrI: contractility index
CV: coefficient of variation
CVD: cardiovascular disease
devP: developed pressure
*dP/dt*_max_: maximum rate of increase in left ventricular pressure per beat
*dP/dt*_min_: peak rate of decrease in left ventricular pressure per beat
DSI: DataSciences International
ECG: electrocardiogram
EjeT: ejection time
ELISA: enzyme-linked immunosorbent assay
EMC: electromechanical coupling time
ENMs: engineered nanomaterials
EP: epinephrine
GAPDH: glyceraldehyde-3-phosphate dehydrogenase
GRK2: G-receptor kinase 2
GRKs: G-protein receptor kinases
GSD: geometric standard deviation
HEPA: high efficiency particulate air
HF: high frequency power spectral heart rate variability
HO-1: heme-oxygenase 1
HR: heart rate
HRV: heart rate variability
HSPH: Harvard T.H. Chan School of Public Health
IACUC: Institutional Animal Care and Use Committee
LF: low frequency power spectral HRV
LQT: Long QT
LQT1: Long QT Syndrome 1
LV: left ventricle or left ventricular
LVEDP: left ventricular end diastolic pressure
LVESP: left ventricular end systolic pressure
LVP: left ventricular pressure
MAO: monoamine oxidase
maxSP: maximum systolic pressure
MN: metanephrine
MRM: multiple reaction monitoring
NE: norepinephrine
NMN: normetanephrine
PAHs: polycyclic aromatic hydrocarbons
Pdur: P-wave duration
PEGS: printer exposure generation system
PEPs: printer emitted particles
PM: particulate matter
pNN15: percentage of pairs of normal RR intervals with > 15 ms difference
ppb: parts per billion
proANP: pro-peptide of atrial natriuretic pepitide
QT: Q-Tend interval
QTc: corrected QT
QTp: Q-Tpeak interval
RMSSD: root mean squared of successive differences in RR intervals
RPM: rotations per minute
RT: relaxation time
RV: right ventricle or right ventricular
Samp: S amplitude
SDNN: standard deviation of normal RR intervals
ST neg area: negative ST area
Tamp: T amplitude
T_co_: core body temperature
tVOCs: total volatile organic compounds
UPLC-MS/MS: ultrahigh performance liquid chromatography with tandem mass spectrometry
VMA: vanillylmandelic acid
VOCs: volatile organic compounds
VPBs: ventricular premature beats
β1AR: β_1_-adrenergic receptors.
