# Supplementary material for: Inhalation of printer-emitted particles impairs cardiac conduction, hemodynamics, and autonomic regulation and induces arrhythmia and electrical remodeling in rats
Source: Part Fibre Toxicol. 2020 Jan 29;17:7. doi: 10.1186/s12989-019-0335-z (PMC6990551; doi:10.1186/s12989-019-0335-z)
Supplement: Supplementary file 1 — Additional file 1: Results. Table S1. Baseline characteristics of treatment groups. Table S2. Daily effects of PEPs exposure on LV pressure, HRV, and ECG morphology. Table S3. Daily effects of PEPs on LV pressure, HRV, and ECG morphology at 2 days, 35 days, and 70 days after exposure cessation, and before, during, and after individual 20-min stress tests. Table S4. Plasma ELISAs results. Table S5. Summary of physiologic effects of PEPs on cardiac hemodynamic, autonomic, and electrophysiologic function. Figure S1. Aerosol characterization over the 21-day exposure to laser printer-emitted particles (PEPs). Figure S2. Effects of PEPs on high frequency HRV during and immediately after exposure. Figure S3. Effects of PEPs on maximum LV pressure upslope (dP/dtmax ), minimum downslope (dP/dtmin ), and rate-pressure product. Figure S4. BRS slope during inhalation exposures. Figure S5. Changes in heart rate and LV pressure before, during, and after 20-min ice water stress tests at 2 days, 5 weeks, and 10 weeks after PEPs. Figure S6. Influence of PEPs on stress-induced ventricular premature beats. Figure S7. Effects of PEPs exposure and ice water stress on core body temperature before, during, and after ice water stress tests. Figure S8. Urinary norepinephrine analyzed by ELISA. Figure S9. Urine catecholamines at 10 weeks after cessation of PEPs exposure measured by HPLC MS/MS. Figure S10. Ratios of parent compounds to daughter metabolites for assessment of metabolic activity at 10 weeks after cessation of PEPs exposure. Figure S11. Serine phosphorylation of Kv7.1 was not significantly altered by PEPs. Figure S12. PEPs does not significantly affect ventricular phosphorylation of ERK or AKT. Figure S13. Effects of PEPs on β1AR expression in the right and left ventricular myocardium. Figure S14. PEPs did not significantly affect RV cardiac troponin I (cTnI) phosphorylation or total heme-oxygenase 1 (HO-1) expression. Figure S15. ECG and LVP analysis. Methods. [file 12989_2019_335_MOESM1_ESM.docx]

|  |  | n | **Air (n=3-4)** | | **PEPs (n=4)** | |
| --- | --- | --- | --- | --- | --- | --- |
| **LV Pressure** | LVESP (mmHg) | 7 | 121.7 | ± 1.4 | 115.7 | ± 1.9 |
|  | dP/dt_max_ (mmHg/s) | 7 | 6780 | ± 74 | 6273 | ± 130 |
|  | CtrI (s^-1^) | 7 | 114.6 | ± 0.8 | 114.6 | ± 2.0 |
|  | CtrT (ms) | 7 | 40.3 | ± 0.3 | 39.3 | ± 0.1 |
|  | EjeT (ms) | 7 | 30.9 | ± 0.5 | 31.3 | ± 0.6 |
|  | Time to dP/dt_max_ (ms) | 7 | 20.3 | ± 0.3 | 19.2 | ± 0.1 |
|  | RT (ms) | 7 | 47.4 | ± 0.3 | 48.3 | ± 0.3 |
|  | FilT (ms) | 7 | 63.7 | ± 2.4 | 62.3 | ± 2.7 |
|  | LVEDP (mmHg) | 7 | 3.6 | ± 0.5 | 3.2 | ± 0.9 |
|  | dP/dt_min_ (mmHg/s) | 7 | -5454 | ± 79 | -5131 | ± 142 |
|  | tau (ms) | 7 | 6.7 | ± 0.1 | 6.8 | ± 0.1 |
|  | devP (ms) | 7 | 125.9 | ± 1.1 | 119.8 | ± 2.4 |
| **Composite intervals** | BRS (ms/mmHg) | 8 | 1.69 | ± 0.14 | 2.16 | ± 0.09 |
|  | RPP (mmHg*beats/min) | 8 | 39209 | ± 190 | 38617 | ± 191 |
|  | EMC (ms) | 8 | 6.04 | ± 0.24 | 6.04 | ± 0.21 |
| **Heart Rate Variability (HRV)** | HR (beats/min) | 8 | 333 | ± 4 | 332 | ± 6 |
|  | RR (ms) | 8 | 181 | ± 2 | 182 | ± 3 |
|  | SDNN (ms) | 8 | 8.10 | ± 0.40 | 11.74 | ± 0.71* |
|  | RMSSD (ms) | 8 | 3.00 | ± 0.34 | 4.68 | ± 0.46 |
|  | CV (1000*ms/ms) | 8 | 4.50 | ± 0.22 | 6.47 | ± 0.35* |
|  | pNN15 (%) | 8 | 0.54 | ± 0.34 | 2.17 | ± 0.59 |
|  | LF (ms^2^) | 8 | 0.60 | ± 0.13 | 1.98 | ± 0.36 |
|  | HF (ms^2^) | 8 | 0.75 | ± 0.22 | 1.74 | ± 0.31 |
|  | LF/HF | 8 | 1.06 | ± 0.14 | 1.28 | ± 0.15 |
| **ECG Morphology** | Pdur (ms) | 8 | 20.5 | ± 0.4 | 19.2 | ± 0.6 |
|  | PR (ms) | 8 | 45.0 | ± 0.6 | 44.8 | ± 0.8 |
|  | QRS (ms) | 8 | 18.2 | ± 0.2 | 18.4 | ± 0.3 |
|  | QTp (ms) | 8 | 29.6 | ± 0.3 | 31.1 | ± 0.6 |
|  | QT (ms) | 8 | 55.6 | ± 1.5 | 56.2 | ± 1.5 |
|  | QTc (ms) | 8 | 57.2 | ± 1.6 | 57.5 | ± 1.4 |
|  | ST neg area (ms*mV) | 8 | -0.78 | ± 0.08 | -0.74 | ± 0.09 |
|  | S amplitude (mV) | 8 | -0.27 | ± 0.02 | -0.21 | ± 0.02 |
|  | T amplitude (mV) | 8 | 0.055 | ± 0.010 | 0.055 | ± 0.014 |

**Additional file 1**

**Results**

**Table S1. Baseline characteristics of treatment groups.** Means ± SEM across four baseline exposure days during the air-exposure phase of monitoring. LV: left ventricular; LVESP: LV end systolic pressure; CtrI: contractility index; CtrT: contractility time; EjeT: ejection time; RT: relaxation time; FilT: filling time; LVEDP: LV end diastolic pressure; devP: developed pressure; BRS: spontaneous baroreflex sensitivity slope; EMC: electromechanical coupling time; SDNN: standard deviation of normal RR intervals; RMSSD: root means squared of successive differences; CV: coefficient of variation; pNN15: percentage of pairs of normal RR intervals with >15 ms difference; LF: low frequency; HF: high frequency; Pdur: P-duration; QTp: Q-T_peak_ interval; QT: Q-T_end_ interval; QTc: corrected QT (*see Methods*); ST neg area: ST negative area. *significantly different from Air group (P<0.05).

|  | **Day 1** | | **Day 5** | | **Day 9** | | **Day 13** | | **Day 17** | | **Day 20** | | **Day 21** | |
| --- | --- | --- | --- | --- | --- | --- | --- | --- | --- | --- | --- | --- | --- | --- |
|  | **Expo** | **Post** | **Expo** | **Post** | **Expo** | **Post** | **Expo** | **Post** | **Expo** | **Post** | **Expo** | **Post** | **Expo** | **Post** |
| **LVP** | | | | | | | | | | | | | | |
| devP (Δms) ^a^ | - | - | - | - | - | **-** | - | - | - | - | - | - | - | **14.7±6.7*** |
| Contr. Time (Δms) ^a^ | - | - | - | - | - | - | - | - | - | - | - | - | **-1.4±0.6*** | - |
| Time to dP/dt_max_ (Δms) ^a^ | - | - | - | - | - | - | - | - | - | - |  |  | **-1.4±0.6*** | - |
| EMC (Δms) | - | - |  |  | - | - |  |  |  |  | - | - | - | **-1.6±0.6*** |
| *no daily differences from Air in change in Filling Time^a^* | | | | | | | | | | | | | | |
| **HRV** | | | | | | | | | | | | | | |
| CV (Δ1000*ms/ms) | - | - | - | - | - | - | - | - | - | - | - | - | **-1.5±0.7*** | - |
| pNN15 (Δ#) | - | - | - | - | - | - | - | - | - | - | - | - | **-2.8±1.1*** | - |
| **ECG morphology** | | | | | | | | | | | | | | |
| Pdur (Δms) | - | **-1.7±0.7*** |  |  | -2.2±1.2 | **-2.8±0.7*** |  |  |  |  |  |  | - | - |
| T amplitude (ΔmV) | - | -0.031±0.016 |  |  | - | *-* |  |  |  |  |  |  | - | *-* |
| *no daily differences from Air in change in PR, QRS, QT ^b^, QTc ^b^, ST negative area ^b^, S amplitude, and T amplitude* | | | | | | | | | | | | | | |

**Table S2. Daily effects of PEPs exposure on LV pressure, HRV, and ECG morphology.** All parameters were assessed as animal-matched change from the average of a 4-day baseline exposure clean air and values are presented as difference from time-matched Air control where P < 0.10, with * and bold font denoting significant difference from Air group (P<0.05). Gray areas indicate days for which data were not analyzed. N=4/group except for ^a^, where n=3 for Air group. ^b^ denotes Interval terminus measured as T-peak.

|  | **2 days post-PEPs** | | | **5 weeks post-PEPs** | | | **10 weeks post-PEPs** | | |
| --- | --- | --- | --- | --- | --- | --- | --- | --- | --- |
|  | **Pre-Stress** | **Stress** | **Post-Stress** | **Pre-Stress** | **Stress** | **Post-Stress** | **Pre-Stress** | **Stress** | **Post-Stress** |
| **LV Pressure** | | | | | | | | | |
| LVESP (ΔmmHg) ^a^ | - | - | - | 7.9±3.6 | 10.7±5.2 | - | **10.2±3.6*** | - | - |
| Ejection Time (Δms) | - | **-7.2±3.2*** | - | **-4.3±1.8*** | -4.6±2.4 | - | **-4.6±1.8*** | -5.3±2.4 | -5.5±3.0 |
| Time to dP/dt_max_ (Δms)^a^ | - | - | - | - | 6.1±3.1 | - | - | - | - |
| tau (Δms) ^a^ | - | - | - | - | - | - | **-0.58±0.19*** | **-0.50±0.20*** | **-0.53±0.20*** |
| Relaxn. Time (Δms) | - | -5.7±3.1 | - | - | - | - | - | - | - |
| dP/dt_max_ (ΔmmHg/s) ^a^ | - | - | - | **848±361*** | 1115±583 | **1094±431*** | **850±361*** | - | - |
| dP/dt_min_ (ΔmmHg/s) ^a^ | - | - | - | **-788±333*** | -1178±572 | **-1070±375*** | **-947±333*** | - | -779±375 |
| devP (ΔmmHg) ^a^ | - | - | - | - | 19.6±10.1 | **16.5±6.0*** | - | - | - |
| *no daily differences from Air in change in CtrI^a^, LVEDP ^a^, and EMC* | | | | | | | | | |
| **HRV** | | | | | | | | | |
| LF (Δms^2^) | - | - | 3.4±1.9 | - | - | - | - | - | - |
| *no daily differences from Air in change in heart rate, SDNN, RMSSD, CV, pNN15, HF, and LF/HF* | | | | | | | | | |
| **ECG** | | | | | | | | | |
| Pdur (Δms) | - | - | - | - | 3.7±1.8 | - | - | - | - |
| S amplitude (ΔmV) | - | - | - | - | 0.059±0.028 | - | - | - | - |
| *no daily differences from Air in change in PR interval, QRS, Q-T_peak_, and heart rate-corrected Q-T_peak_* | | | | | | | | | |

**Table S3.** **Daily** **effects of PEPs on LV pressure, HRV, and ECG morphology at 2 days, 35 days, and 70 days after exposure cessation, and before, during, and after individual 20-min stress tests.** All parameters were assessed as animal-matched change from the average of a 4-day baseline exposure to clean air and values are presented as difference from time-matched Air control where P < 0.10, with * and bold font denoting significant difference from Air group (P<0.05). N=4/group except for ^a^, where n=3 for Air group. ^c^: Interval terminus measured as T-peak. “-“ indicates no statistically differences or trends of differences from Air. BRS was not analyzed on these days. For effects on additional repolarization parameters, see *Figure 9*.

| **Plasma ELISAs (ng/mL)** | | | | | | |  |
| --- | --- | --- | --- | --- | --- | --- | --- |
|  | **Air** | | **PEPs** | | **P-value** |  |  |
| BNP-45 | 1.78 | ± 0.82 | 1.01 | ± 0.42 | 0.43 |  |  |
| cTnI | 0.004 | ± 0.002 | 0.021 | ± 0.013 | 0.24 |  |  |
| proANP | 0.305 | ± 0.077 | 0.264 | ± 0.032 | 0.44 |  |  |

**Table S4. Plasma ELISAs results.** EDTA-collected plasma was assayed using commercially available ELISA kits for B-type natriuretic peptide 45 (BNP-45), cTnI, and pro-peptide of atrial natriuretic peptide (proANP). Two-tailed t-tests were performed for comparisons. Means ± standard error, n=4/group.

| Physiologic Trait | Parameter | **PEPs Exposure** | | **post-PEPs Stress Test** | | |
| --- | --- | --- | --- | --- | --- | --- |
|  |  | Mid- | Early Post- | Pre-Stress | Mid-Stress | Post-Stress |
| Afterload | ESP | ↑d21 | ↑ | ↑↑ |  |  |
| Contractility | CtrI | ↓ |  |  |  |  |
|  | EjeT | ↓↓ | ↓↓ | ↓ | ↓↓ | ↓ |
| Preload | EDP |  |  |  |  |  |
| Lusitropy | *Tau*^-1^ | ↑↑ | ↑↑ | ↑ | ↑70d | ↑70d |
|  | RT^-1^ | ↑↑ | ↑↑ |  |  | ↑↑ |
| Chronotropy | Heart rate |  |  | ↑ |  |  |
| Workload | RPP | ↑d1 | ↑d20-d21 |  |  |  |
| Relative Parasympathetic Influence | SDNN | ↓d21 |  |  |  |  |
|  | RMSSD | ↓↓ | ↓ |  |  |  |
|  | HF | ↓d21 |  |  |  |  |
|  | (LF/HF)^-1^ |  | ↓↓ |  |  |  |
| Baroreflex Sensitivity | BRS slope | ↓ |  |  |  |  |
| Atrial Depolarization Time | P dur | ↓↓ | ↓↓ |  |  |  |
| Ventricular Depolarization Time | S amplitude | ↓↓ |  |  |  |  |
| Ventricular Repolarization Time | QT |  | ↑ |  | ↑↑^pre^ |  |
|  | QTc |  | ↑↑ |  | ↑↑ ^pre^ | ↑↑ ^pre^ |
|  | ST neg area |  |  |  |  | ↑↑ ^pre^ |
|  | T amplitude |  |  |  | ↑↑ ^pre^ | ↑↑ ^pre^ |
|  | TpTe |  |  |  | ↑↑ ^pre^ |  |
|  | TpTe/QT |  |  |  | ↑↑ ^pre^ | ↑2d |
| Arrhythmia | VPBs |  |  | ↑2d-35d |  |  |
| Thermoregulation | Core Temperature |  |  | ↑↑ | ↑35d-70d | ↑35d-70d |

**Table S5.** **Summary of** **physiologic** e**ffects of PEPs on cardiac hemodynamic, autonomic, and electrophysiologic function.** Large arrows denote differences between groups across entirety of observation period (overall), for which double arrows are statistically significant (P<0.05) and single arrows are trends (P<0.10). Small arrows indicate significant daily differences (P<0.05). ^-1^ indicates parameter was inverted to positively reflect physiologic trait. ^pre^ indicates parameter differed between groups according to change from same-day pre-stress value. Gray boxes indicate parameter not evaluated at experimental timepoint.

**Figure S1. Aerosol characterization over the 21-day exposure to laser printer-emitted particles (PEPs).** Measured by SMPS with a cutoff point of 162.5 nm. (A) Mean particle number and mass concentrations (± SD) as a function of mobility diameter of PEPs. (B) Daily mean particle diameter and count median diameter during the PEPs exposure in each experiment group. (C) Daily particle number and mass concentrations during the PEPs exposure in each experiment group (mean ± SD).

**Figure S2. Effects of PEPs on high frequency HRV during and immediately after exposure.** Values calculated as mean (± standard error) of each animal’s change from its 4-day baseline (mid-expo = 5 h/day; post-expo = (30 min/day). n=4/group. Day +1 marks post-exposure day in monitoring cages instead of exposure chambers. *P < 0.05 vs. Air. LF did not differ between groups at mid- or post-expo.

 **Figure S3. Effects of PEPs on maximum LV pressure upslope (dP/dt_max_ ), minimum downslope (dP/dt_min_ ), and rate-pressure product.** Values calculated as mean (± SEM) of each animal’s change from its 4-day baseline (mid-expo = 5 h/day; post-expo = 30 min/day. PEPs n=4, Air n=3. Day +1 marks post-exposure day in monitoring cages instead of exposure chambers. *P < 0.05 vs. Air.

**Figure S4. BRS slope during inhalation exposures.** Values are expressed as mean change from animal-matched exposure baseline (± SEM). N=4/group. There was an overall trend of decreased BRS during exposure, but no individual day differences. ^#^P < 0.10 and *P < 0.05 vs. Air.

**Figure S5. Changes in heart rate and LV pressure before, during, and after 20-min ice water stress tests at 2 days, 5 weeks, and 10 weeks after PEPs.** Values are mean (± SEM) of change from 4-day baseline (BL) at 2, 35, and 70 days after cessation of inhalation exposures and immediately before, during, and after stress tests (20 min each). PEPs n=4, Air n=3. ^#^P < 0.10 and *P < 0.05 vs. Air.

**
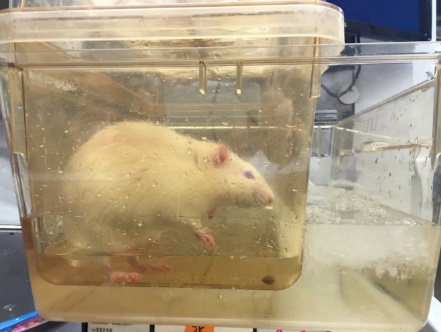
**

Internal cage water line (2 cm deep)

External tank water line (for insulation)

 **Figure S6. Influence of PEPs on stress-induced ventricular premature beats.** Ventricular premature beats (VBPs) during and after ice water stress tests. **Upper panel**, experimental setup for ice water stress test. **Lower panel**, frequency of VPBs during and after stress test. Values expressed as mean (± SEM) count of VPBs per hour during 20-min observation periods. N=4/group. ^#^P = 0.05 and *P < 0.05 vs. Air.

**Figure S7. Effects of PEPs exposure and ice water stress on core body temperature before, during, and after ice water stress tests.** Means ± mean ± SEM of either raw values (Pre-Stress, 20 min) or changes from immediately prior (Pre-Stress, Δ) at 2, 35, and 70 days after cessation of inhalation exposures during Mid- and Post-Stress periods (20 min each) for each group, n=4/group. ^#^P < 0.10 and *P < 0.05 vs. Air. Differences within treatment group relative to specific prior monitoring day are indicated above bars (e.g., “2d”).

**Figure S8. Urinary norepinephrine analyzed by ELISA.** Circles indicate individual rat values, with lines denoting mean ± SEM. N=4/group.

**Figure S9. Urine catecholamines at 10 weeks after cessation of PEPs exposure measured by HPLC MS/MS.** Urine was collected overnight before the stress test and again overnight after the stress test**.** Circles indicate individual rat values, with lines denoting mean ± SEM, normalized by urinary creatinine. N=4/group. * indicates P < 0.05.

**Figure S10. Ratios of parent compounds to daughter metabolites for assessment of metabolic activity at 10 weeks after cessation of PEPs exposure.** Urine was collected overnight before the stress test and again overnight after the stress test. Circles indicate individual rat values, with lines denoting mean ± SEM, normalized by urinary creatinine.

 **Figure S11. Serine phosphorylation of K_v_7.1 was not significantly altered by PEPs.** Values represent mean ratio of serine-phosphorylated K_v_7.1 to total immunoprecipitated K_v_7.1 (±SEM ) assessed by Western blot. Tissues were harvested at 10 weeks post-exposure to filtered air or PEPs. Circles indicate individual rat values, with lines denoting mean ± SEM, n=4/group.

**Figure S12. PEPs does not significantly affect ventricular phosphorylation of ERK or AKT.** Tissues were harvested at 10 weeks post-exposure to filtered air or PEPs. Circles indicate individual rat values, with lines denoting mean ± SEM, n=4/group.

PEPs exposure corresponded with an apparent 55% decrease in phosphorylated ERK1/2 in the RV that was not statistically significant (Fig. S12; P = 0.19), whereas no effect of PEPs on phosphorylation of LV ERK was observed (P=0.85 vs. Air).

**Figure S13. Effects of PEPs on β_1_AR expression in the right and left ventricular myocardium.** Normalized to GAPDH. RV:LV represents the group mean ratio of ventricular protein densities (GAPDH-normalized) for each rat. Circles indicate individual rat values, with lines denoting mean ± SEM, n=4/group.

PEPs also increased RV β_1_AR expression by 58%, but this effect was not significant upon normalization by GAPDH (Fig. S13; P=0.11 vs. Air). To determine potential desensitization of β_1_AR, we assayed RV G-receptor Kinase 2 (GRK2) expression by immunoblot and saw no effect of PEPs (P=0.81 vs. Air, data not shown). Additionally, we assessed the expression of myostatin and phosphorylation of Akt given their association with cell cycle arrest and glucose uptake [64, 65]. PEPs did not significantly affect phosphorylation of Akt on Thr308 (Fig. S12) or expression of myostatin in either ventricle (not shown).

**Figure S14. PEPs did not significantly affect RV cardiac troponin I (cTnI) phosphorylation or total heme-oxygenase 1 (HO-1) expression.** Tissues were harvested at 10 weeks post-exposure to filtered air or PEPs. Circles indicate individual rat values, with lines denoting mean ± SEM, n=4/group.

Per apparent increases in β_1_AR expression and decreases in ERK phosphorylation, we also measured for RV cardiac troponin I phosphorylation (Ser23,24) given its central role in β_1_AR-dependent increases in diastolic function, and heme-oxygenase 1 expression due to its close ties to inflammation and oxidative stress, and neither were significantly affected (Fig. S14). Finally, exposure to PEPs was not associated with any treatment-related differences in circulating BNP-45, proANP, or cTnI (Table S4).


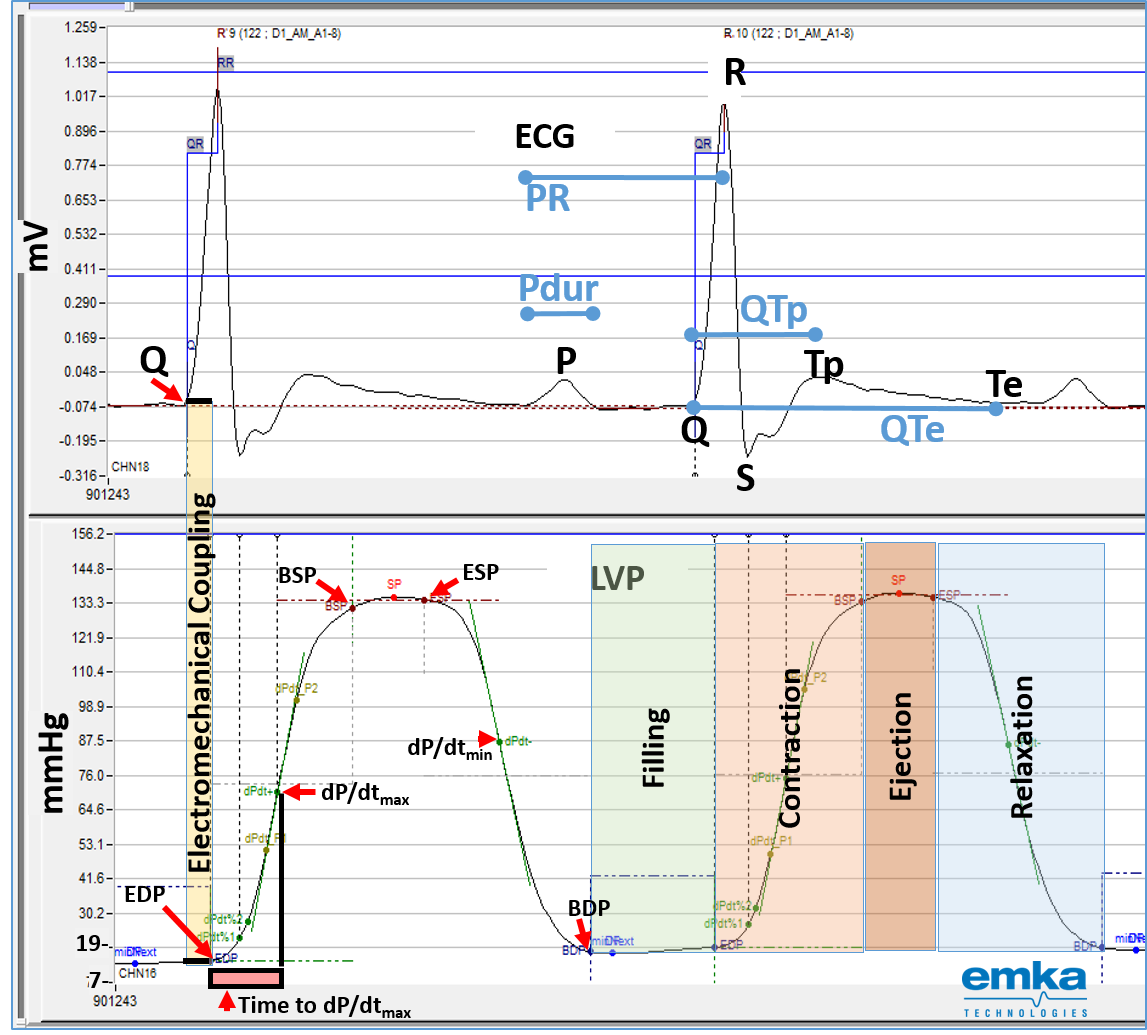


**Figure S15. ECG and LVP analysis.** Representative rat ECG waveform showing basic fiducial points and endpoints, including QT interval (QTe, time from Q to T-end), PR interval (PR), and P duration (Pdur). P and Tp denote P_peak_ and T_peak_ respectively. Te denotes T_end_. For assessment of QT interval, QTe was used on stress test days, whereas QTp was used at baseline and on inhalation exposures days.

**Methods**

**Animals and surgery for telemeter implantation.** Rats were injected with Meloxicam (1 mg/kg *s.c.*) and anesthetized with isoflurane (induced with 5% at 1 L/min oxygen and maintained with 1-2% at 0.5 L/min oxygen), tracheally intubated, connected to a ventilator (70 breaths/min, 1.8 mL/breath; Harvard Apparatus), and placed in dorsal recumbency on a heating pad. The abdomen was shaved and disinfected, and incised ≈5 cm along the linea alba to the xiphoid cartilage. After the liver was retracted using moist gauze, the diaphragm was cut (≈1 cm) to visualize the LV, which was retracted by anchor suture, punctured with a 21-gauge needle, and inserted with a 2-F catheter secured to the LV by purse-string suture (7-0 Deklene). The diaphragm was sealed by 5-0 vicryl suture and Vetbond (3M), the animal was removed from intubation, and the chest was evacuated of all air to resume negative pressure. The esophagus was then isolated, retracted with 5-0 silk suture, and the serosal layer punctured with a 20-gauge IV catheter. A second pressure catheter was inserted into the puncture and advanced to obtain an optimal signal. The suture retraction was released to ensure correct placement and the pressure catheter was glued in place using Vetbond. The suture was removed and a fiber patch was glued over the catheter /esophagus junction with Vetbond. The transmitter was inserted into the abdominal cavity and the ECG leads exteriorized through the abdominal wall. The abdominal incision was closed with 4-0 suture and the ECG leads were subcutaneously sutured to the right pectoral muscle region (negative) and the left caudal rib region (positive), approximating a Lead II configuration. Skin incisions were closed with wound clips and Gluture, and the animal was recovered in a cage placed halfway on a heating pad for 24 hours.

**Immunoprecipitation of K_v_7.1.** Pierce Classic IP kit (Cat. # 26146) was used. Left and right ventricular homogenates of heart tissue in 1X RIPA buffer (50mM Tris, 150mM NaCl, 1% Triton X-100, 0.25% Sodium deoxycholate, 0.1% SDS, 1mM EDTA, pH 7.4) were incubated in the spin column containing resin with gentle rocking at 4℃ for an hour. After centrifugation of the column, K_v_7.1 antibody (3µg/mL concentration) (Alomone Labs, Cat. # APC-022) was added to the flow through and incubated overnight at 4℃ to form immune complex. The immune complex was added to the resin in spin column and incubated for an hour on a rocker. The resin was washed three times with wash buffer and finally with conditioning buffer. Following sample buffer elution, sample was analyzed by Western Blotting for phosphoserine (Abcam #9332) and K_v_7.1 (Alomone).
